# Pose Generation for Social Robots in Conversational Group Formations

**DOI:** 10.3389/frobt.2021.703807

**Published:** 2022-01-17

**Authors:** Marynel Vázquez, Alexander Lew, Eden Gorevoy, Joe Connolly

**Affiliations:** Department of Computer Science, Yale University, New Haven, CT, United States

**Keywords:** human–robot interaction (HRI), group conversations, F-Formations, spatial behavior analysis, proxemics

## Abstract

We study two approaches for predicting an appropriate pose for a robot to take part in group formations typical of social human conversations subject to the physical layout of the surrounding environment. One method is model-based and explicitly encodes key geometric aspects of conversational formations. The other method is data-driven. It implicitly models key properties of spatial arrangements using graph neural networks and an adversarial training regimen. We evaluate the proposed approaches through quantitative metrics designed for this problem domain and via a human experiment. Our results suggest that the proposed methods are effective at reasoning about the environment layout and conversational group formations. They can also be used repeatedly to simulate conversational spatial arrangements despite being designed to output a single pose at a time. However, the methods showed different strengths. For example, the geometric approach was more successful at avoiding poses generated in nonfree areas of the environment, but the data-driven method was better at capturing the variability of conversational spatial formations. We discuss ways to address open challenges for the pose generation problem and other interesting avenues for future work.

## 1 Introduction

In this work, we study how to generate appropriate poses for social robots to take part in conversational group formations with users. This problem is important because people naturally establish these spatial formations with social robots when conversing with them (Hüttenrauch et al., 2006; Kuzuoka et al., 2010; Vázquez et al., 2015a; Karreman et al., 2015; Bohus et al., 2017). Further, people expect robots to conform to these formations when adapting to changes to group members (Vázquez et al., 2017; Yang et al., 2017).

**FIGURE 1:**
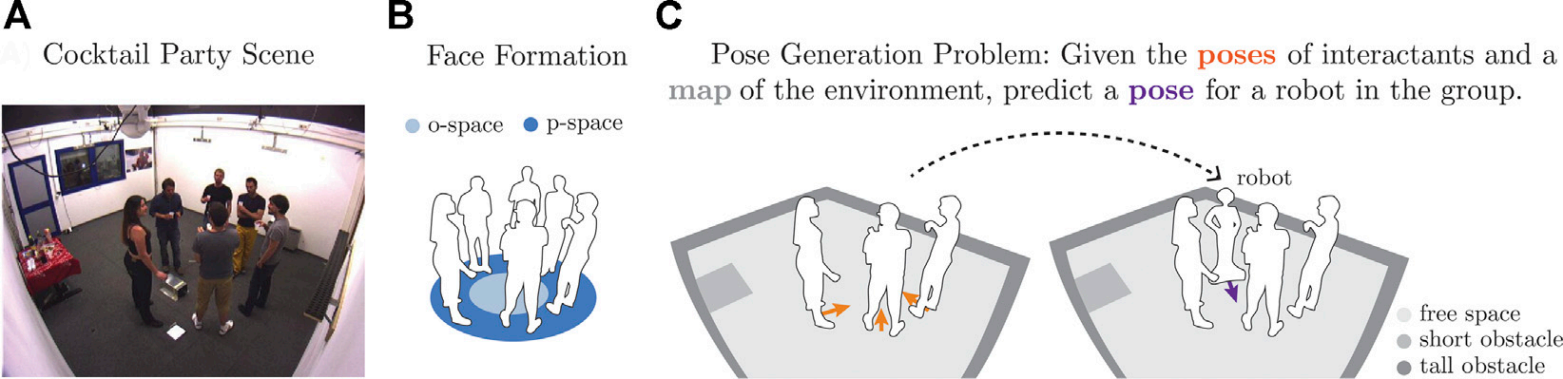
Conversational spatial arrangement from the Cocktail Party dataset **(A)**, spatial formation typical of group conversations **(B)**, and problem setup **(C)**.

Although it is common to model conversational spatial behavior with discriminative models of group formations (Truong and Ngo, 2017; Vázquez et al., 2017; Hedayati et al., 2019; Barua et al., 2020; Swofford et al., 2020), we approach the problem of predicting a pose for a robot in a group conversation with generative models. These models can directly output poses for the robot based on the social context of the interaction and spatial constraints imposed by the environment, for example, due to small objects such as tables or bigger structures such as walls. An illustrative example is provided in Figure 1.

In this work, we explore two approaches for generating spatial behavior: a model-based, geometric approach that explicitly encodes important properties of conversational group formations as often discussed in the social psychology literature (Kendon, 1990), and a data-driven adversarial approach that, once trained, implicitly encodes these properties. While our geometric approach builds directly in some cases on prior work, to the best of our knowledge, no prior effort has explored generating suitable spatial behavior for conversations subject to spatial constraints due to the environment layout. By studying these two methods, this work contributes not only novel approaches, but also better understanding of how model-based and data-driven solutions for spatial reasoning in Human–Robot Interaction (HRI) complement each other.

We evaluated the proposed approaches quantitatively and qualitatively in relation to expected spatial behavior. Also, we conducted an online evaluation to gather human opinions about each method’s performance when applied to situated human-robot interactions. Our results show that incorporating spatial constraints into models for pose generation is beneficial. Further, we show that the proposed methods can be used to effectively model a nonparametric distribution of poses for conversational groups. In practice, we find that considering this distribution when generating an appropriate pose can lead to better results than predicting a single pose directly. Interestingly, the human evaluation suggested that the geometric approach was more effective than the data-driven method when applied to small groups such as dyadic interactions, but the data-driven approach was better for groups with four to six interactants. We discuss ways to address this disparity. Lastly, we demonstrate the applicability of the proposed approaches for simulating conversational spatial arrangements.

## 2 Background

Before explaining how the proposed methods work, the next sections provide a brief introduction to conversational group formations from a social psychology perspective and introduce Graph Neural Networks (GNNs) from a message-passing point of view. The former description is important for contextualizing the proposed geometric approach for pose generation and for understanding the rationale behind several of the metrics used in our evaluation. The latter primer on GNNs aids in understanding the proposed data-driven approach.

### 2.1 Conversational Group Formations

During human conversations, people often position and orient themselves in special spatial patterns known as Face Formations (F-Formations) (Kendon, 1990). F-Formations are characterized by people being nearby one another such that they can communicate easily. Also, interactants tend to direct their lower bodies toward one another or toward a common focus of attention for the conversation. These behaviors lead to spatial arrangements where individuals typically have equal, direct, and exclusive access to a common space. The formations keep groups as separate units from other close interactions.

Figure 1A depicts an example F-Formation from the Cocktail Party dataset (Zen et al., 2010), a computer vision dataset that is often used for evaluating group detection approaches based on human spatial behavior (Ricci et al., 2015; Setti et al., 2015). As illustrated in Figure 1B, the interior region of an F-Formation is known as its *o-space*. The area where people stand around the o-space is the *p-space*. Later in this article, we refer to these terms when formally describing geometric properties of F-Formations.

### 2.2 Graph Neural Networks

In this work, we use the message-passing framework for GNNs proposed by Gilmer et al. (2017), Battaglia et al. (2018) to design our data-driven pose generator method. In contrast to more traditional algorithms for reasoning about graphs, GNNs allow for learning representations, the structure of entities, and relations from graph data. Consider a graph G=(u,V,E) , where the vector **u** is a global attribute (or feature) for the graph, the set $V={\left\{v_{i}\right\}}_{i=1:n}$ corresponds to features for the graph’s vertices, and $E={\left\{\left(e_{k},r_{k},s_{k}\right)\right\}}_{k=1:m}$ is the set of edge features **e**_*k*_ with (*r*_*k*_, *s*_*k*_) being the indices of the nodes connected to the edge. Then, a Graph Network block (GN block)—the basic element of a GNN—can be used to transform a graph G into an updated graph $G^{\prime }=\left(u^{\prime },V^{\prime },E^{\prime }\right)$ via three steps. First, the edge features are updated. Second, the node features are updated, potentially using aggregated edge information. Third, the global attribute for the graph is updated, perhaps using node and edge information as well. Because these operations are implemented via differentiable functions, as further detailed below, the GN block can be integrated as a module into more complex neural network models.

In this work, we are concerned with using GNNs to compute vector representations for fully connected social interaction graphs that describe conversations. These graphs have a global attribute **u**, corresponding to contextual information for the interaction, such as the layout of the physical environment. The graphs’ node features encode pose information for the interactants, but they have no relevant edge features. Thus, applying the GN block computation to them consists of two main steps: updating the nodes features and then updating the global graph attribute. The updated global graph attribute is used to represent the graph in downstream tasks. Mathematically, we can express the two key GN block operations as follows:

$$\begin{array}{ll} v_{i}^{\prime } & =\phi^{v}\left(v_{i}\right) \end{array}$$ (1)

$$\begin{array}{ll} \overset{¯}{v}\prime & =\rho^{v\to u}\left({\left\{v_{i}^{\prime }\right\}}_{i=1:n}\right) \end{array}$$ (2)

$$\begin{array}{ll} u^{\prime } & =\phi^{u}\left(\bar{e}\prime ,\bar{v}\prime ,u\right) \end{array}$$ (3)

where the *update* functions *ϕ* ^*v*^(⋅), *ϕ* ^*u*^(⋅) and the *aggregate* function *ρ* ^*v*→*u*^(⋅) are differentiable functions. In general, the aggregate function should take a variable number of arguments so that the GN block is suitable for processing different graphs. In addition, *ρ* ^*v*→*u*^(⋅) is often implemented as a symmetric mathematical function (such as element-wise summations or maximum) because it is common for graph nodes to lack a natural order. Note that Equations 1–3 are similar to deep set operations (Qi et al., 2017; Zaheer et al., 2017). Indeed, deep sets are sometimes regarded as a specialization of GNNs (Battaglia et al., 2018).

## 3 Related Work

Experimental HRI work has validated the idea that spatial formations typical of human–human conversations naturally emerge in human-robot interactions (Hüttenrauch et al., 2006; Kuzuoka et al., 2010; Karreman et al., 2015; Vázquez et al., 2015a, 2017). In turn, this research led to work on recognizing F-Formations in robotics, such as methods geared toward improving robot navigation (Rios-Martinez et al., 2011), generating multimodal nonverbal robot behavior (Vázquez et al., 2017), helping recognize the beginning and ending of human-robot interactions (Gaschler et al., 2012), joining groups (Barua et al., 2020), and other approaches for service robots (Hedayati et al., 2019; Swofford et al., 2020). Oftentimes, prior work on F-Formation detection in robotics builds on mathematical models of human F-Formations from the computer vision community, for example, (Cristani et al., 2011; Setti et al., 2013; Setti et al., 2015; Vascon et al., 2014). In a similar manner, mathematical models from computer vision inspired the proposed geometric approach for generating poses for a robot in a conversation and motivated a variety of evaluation metrics in this work.

Several methods for generating spatial behavior representative of F-Formations have been proposed in HRI. For example, Vázquez et al. (2016) explored reinforcement learning for adapting the pose of a robot during conversations. Morales et al. (2014) proposed a method for a robot to walk side-by-side to a human. In addition, other work has investigated methods for robots to approach F-Formations (Shi et al., 2011; Truong and Ngo, 2017; Yang and Peters, 2019; Yang et al., 2020a). Among these methods, that of Yang and Peters (2019) is closest to our work because they explore generative adversarial networks to predict appropriate robot navigation behavior. Similar to this prior work, we are interested in modeling spatial behavior during group conversations; different to it, though, we make predictions without temporal information and subject to environmental spatial constraints, for example, nearby walls and objects.

Close to our work, Swofford et al. (2020) used a neural network model to detect F-Formations. We build on this effort because we use a similar network architecture to handle variable group sizes. Interestingly, we make an explicit connection between this prior work—which was inspired by deep sets—and GNNs following (Gilmer et al., 2017; Battaglia et al., 2018). It is worth nothing that the idea of representing interactions with graphs (as described in Section 2.2) is inspired by foundational work on detecting F-Formations (Hung and Kröse, 2011; Vascon et al., 2014) and a long history of applications of graph theory to social network analysis (Scott, 1988; Borgatti et al., 2009; Hamilton et al., 2017).

Among prior work that has used GNNs to reason about situated social interactions, that of Yang et al. (2020b) is perhaps the closest prior effort. While their work aimed to classify human behavior in group social encounters, we instead use GNNs to model properties of spatial formations and predict an interactant’s pose within an adversarial neural network framework. Battaglia et al. (2018) and Hamilton (2020) discuss broader applications of GNNs, which are beyond the scope of this paper.

Another important related work is that of Yang et al. (2017), which proposed an approach for a robot to position itself relative to humans during a group conversation. Because this approach builds on geometric properties of F-Formations, we consider it as a baseline for the proposed methods in our evaluation.

There has also been interest in generating appropriate spatial behavior for social agents within the virtual agent community. For example, Jan and Traum (2007) considered the problem of computing agents’ positions in order to create circular group formations. We also consider circular groups in this work, although these arrangements are often idealistic, as shown in our experiments. In addition, Pedica and Högni Vilhjá lmsson (2010) proposed an approach to generate human-like motion for virtual characters based on the territorial organization of social situations, including F-Formation systems. Their approach used a combination of low-level reactive behaviors to control the pose of social avatars as they move in virtual worlds. Similar to this work, we consider social norms as a driving factor when generating spatial behavior for robots and when evaluating the results of the proposed methods. Different to this prior effort, we do not expect a user to provide pose commands for the social agent of interest; rather, we study the problem of automatically generating suitable poses for a robot in a conversation.

## 4 Generating Appropriate Poses During Conversations

We contribute two approaches to generate an appropriate pose for a social robot in a group conversation. The key novelty of these methods stems from considering environmental spatial constraints along with the pose of other interactants upon making a prediction. These methods are both generative models, capable of representing the distributions of suitable poses via a discrete set of samples.

### 4.1 Problem Statement

Consider a social robot in a human environment in which there are other people with whom the robot wants to establish a situated conversation. We formulate the problem of generating an appropriate pose for the robot to sustain the conversation as follows: let *C* = { < **x**_*i*_, *θ*_*i*_ > | 1 ≤ *i* ≤ *P*} be the social *context* of the interaction encoded by the poses of the *P* people with whom the robot wants to converse, where $x_{i}={\left[x_{i}\;y_{i}\right]}^{T}$ is their position on the ground, and *θ*_*i*_ is their body orientation. In addition, let *M* be a metric two-dimensional (2D) *map* with semantic labels for the physical environment surrounding the group. The labels encode the probability of occupancy, such as occupied space by a “small or movable object” or a “tall barrier” like a wall. Then, the goal is to compute a pose $\bar{p}=<x,\theta >$ for the robot to take part in the conversational group given *C* and *M*, as illustrated in Figure 1C. The generated pose should preserve the spatial structure of the group, that is, their F-Formation. Also, the pose should be such that the robot does not collide with objects according to the map, as well as does not violate social norms such as personal space.

### 4.2 A Geometric Approach for Pose Generation

One way to compute a viable pose for a robot to take part in a conversational group is to explicitly formalize key geometric properties of its expected spatial behavior. To this end, we first consider the fact that F-Formations often have a circular shape because of people’s tendency to position in a way such that they can see and monitor one another during conversations (Kendon, 1990). The circular shape not only defines an expected distribution for people’s locations but also guides their body orientations toward the center of their group’s o-space. Second, we consider the fact that the agent should not be in an occupied location and should not violate other people’s personal space.

Based on the above properties, we propose a three-step algorithm for computing a pose $\bar{p}=<x,\theta >$ given the context *C* and map *M*:

1) Fit circular shape to the context poses. We represent the geometric shape of the group formation parametrically with a 2D circle or ellipse fitted to the context *C* (as illustrated in Figures 1A,B). The edge of the shape represents the p-space of the F-Formation, whereas its interior corresponds to the o-space.

Intuitively, fitting an ellipse should be preferable to fitting a circle because of the variability of human spatial behavior. However, we sometimes default to using circles because fitting ellipses requires at least 5 points.To fit a circle, we consider three cases. First, if the context has a single individual, |*C*| = 1, then we assume that the center of the circle is *d* units in front of the individual, in the direction of its transactional segment. This means that the o-space of the group is defined by the circle with a center at $c=x_{1}+d\;{\left[\cos\left(\theta_{1}\right)\;\sin\left(\theta_{1}\right)\right]}^{T}$ and a radius of *d*. The distance *d* has been defined in the literature as the *stride* parameter of mathematical F-Formation models (Cristani et al., 2011). Second, if the context has two individuals, |*C*| = 2, then we assume that the center of their group’s o-space is in between them because face-to-face spatial arrangements are common for dyads. This means that the center of the circle is given by **c** = (**x**_1_ + **x**_2_)/2, and its radius is ‖**x**_1_ − **x**_2_‖/2. Third, if the context has at least three people, |*C*| > = 3, then we fit a circle to their locations using orthogonal distance regression (Boggs and Rogers, 1990), which tends to be more robust to potential errors in the location measurements than ordinary least squares.To fit an ellipse to the location of the interactants in *C*, we follow the direct fitting approach by Halíř and Flusser (1998). We found this approach to be fast in comparison to iterative approaches and more robust than that of Fitzgibbon et al. (1996) when |*C*| = 5.

**FIGURE 2:**
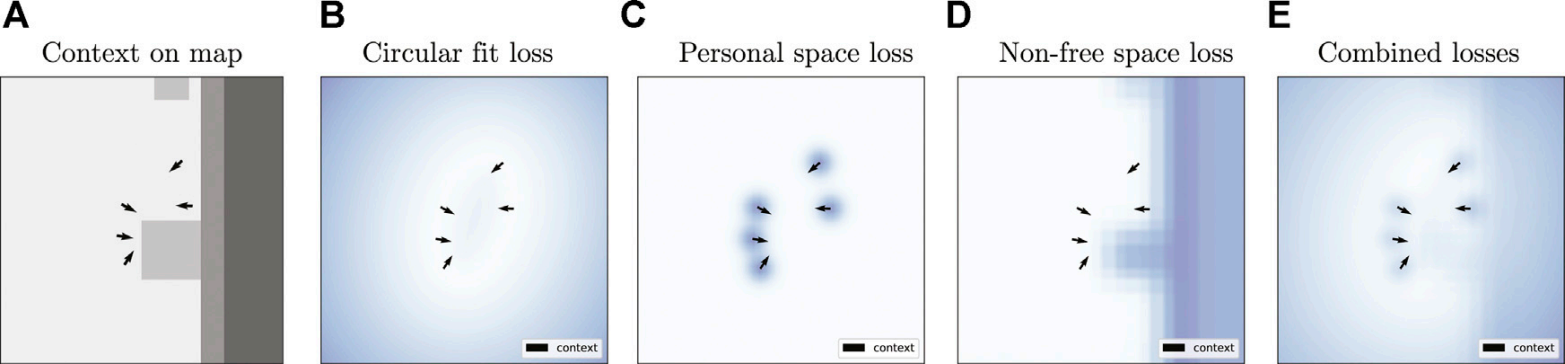
Example losses for the Geometric approach on a sample from the Cocktail Party dataset. From left to right: **(A)** social context on an environment map with free space (light gray color), short obstacles (medium gray), and tall obstacles (darker gray); **(B)** circular fit loss; **(C)** personal space loss; **(D)** penalty associated with nonfree cells of the environment map; and **(E)** weighted sum of those three losses. The arrows indicate the pose of the interactants. Brighter values in the loss plots correspond to lower cost.

2) Compute the robot’s location. We view the problem of computing a suitable location for the robot given the fitted circular shape, the context *C*, and map *M* as an optimization problem. The key factor in this formulation is the loss function, which we define as a weighted sum of three components that penalize for deviations from the fitted circular shape (*ℓ*_*c*_), close proximity to other individuals (*ℓ*_*p*_), and positioning in nonfree areas of the environment (*ℓ*_*f*_). Formally:

$$\ell \left(x\right)=\lambda_{c}\ell_{c}\left(x\right)+\lambda_{p}\ell_{p}\left(x\right)+\lambda_{f}\ell_{f}\left(x\right)$$ (4)

where $\lambda_{c},\lambda_{p},\lambda_{f}\in R^{+}$ control the effect of each penalty. The first component *ℓ*_*c*_ corresponds to the perpendicular distance from **x** to the fitted circle or ellipse. The second component *ℓ*_*p*_ penalizes violations to personal space: $\ell_{p}\left(x\right)=\sum_{i=1}^{P}N\left(x;x_{i},I\sigma \right)$ , where N denotes a normal distribution with mean **x**_*i*_ and variance *σ*. Lastly, *ℓ*_*f*_ in Eq. 4 is a penalty for the input location corresponding to a nonfree cell of the map *M*. Figures 2A–E illustrate these different components for the loss, where the map has been smoothed to avoid positions too close to nonfree cells.

While one could use brute-force search to find a minima of Eq. 4 around the context *C*, we propose to minimize the loss using Powell’s conjugate direction method (Powell, 1964), a popular optimization algorithm. This method does not require derivatives, which is convenient for this optimization because computing the orthogonal distance to an ellipse, as needed by the *ℓ*_*c*_ penalty, is a nontrivial problem for which we use an iterative method. See Uteshev and Yashina (2015) and Uteshev and Goncharova (2018) for a discussion on the point-to-ellipse problem.

3) Compute the robot’s orientation. We finally set *θ* such that the robot orients toward the center of the fitted circular shape, corresponding to the expected center of the o-space.

### 4.3 A Data-Driven Adversarial Approach for Pose Generation

Another way to approach the problem of generating a suitable pose for a robot in a conversation is to leverage generative data-driven methods. In particular, we explore using Wasserstein Generative Adversarial Networks (WGAN), originally proposed by Arjovsky et al. (2017), to produce poses that conform to measured characteristics of F-Formations. This type of data-driven model is composed of two neural networks: a *generator* *G*, which we use to predict the desired pose $\bar{p}$ ; and a *discriminator* *D*, which helps discern generated poses from poses in the true data. Note that for WGANs, *D* is often called the *critic* because the network is not trained to classify, but outputs a real value; here, we use the terms interchangeably to help readers familiar with adversarial networks follow our explanation.

Without loss of generality, let us represent the pose of a social agent < **x**, *θ* > as a 4D row vector **p** = [*x y*  cos(*θ*)  sin(*θ*)] so that we do not have to worry about *θ* wrapping around the (−*π*, *π*] interval. Also, assume that we have a dataset $D=\left\{<C^{j},M^{j},p^{j}>\right\}$ with ideal poses **p** for a social robot given a corresponding context *C* and map *M*. Our goal with the WGAN is to then train the generator and discriminator networks using D . Formally, the WGAN objective can be expressed as a minimax game:

$$\underset{G}{\min}\;\underset{D}{\max}E_{p∼P_{r}}\left[D\left(p|C,M\right)\right]-E_{\bar{p}∼P_{g}}\left[D\left(\bar{p}|C,M\right)\right]$$ (5)

where we have conditioned the discriminator *D* on the corresponding context and map data for the sampled pose, following the formulation for Conditional Generative Adversarial Networks by Mirza and Osindero (2014). The discriminator (or critic) in Eq. 5 should be in the set of 1-Lipschitz functions, which we implement via a gradient penalty added to the loss in Eq. 5 per (Gulrajani et al., 2017). Lastly, $P_{r}$ in Eq. 5 is the real data distribution induced by D , and $P_{g}$ is the distribution implicitly defined by the generator *G*: $\bar{p}=G\left(z|C,M\right)$ , with the latent variable **z** ∼ *p*(**z**) coming from a simple prior (e.g., a standard normal distribution in this work).

**FIGURE 3:**
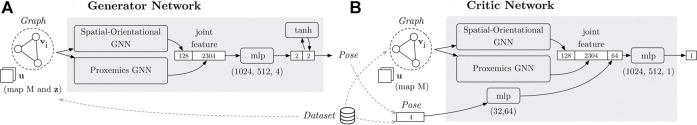
The generator **(A)** and the critic **(B)** process the information in the social interaction graph via two GNNs. One GNN reasons about the spatial–orientational arrangement of the group (encoded in the vertex features **v**_*i*_). The other GNN reasons about proxemics based on the interactant’s positions (encoded in the vertices) and the map (encoded in the global attribute **u**). Note that the global attribute for the graph input to the generator also includes the latent variable **z**. The “mlp” blocks are multilayer perceptrons.

We propose a novel two-stream architecture for the generator and discriminator networks (Figure 3). This architecture is driven by our knowledge of the problem domain—we take advantage of inductive biases (in terms of relational and spatial structure) to facilitate learning. The next sections provide more details.

#### 4.3.1 The Generator Network

Figure 3A describes how the generator predicts a pose $\bar{p}$ given a social interaction graph G as input. The nodes of the graph correspond to pose features **v**_*i*_ = [*x*_*i*_ *y*_*i*_   cos(*θ*_*i*_)  sin(*θ*_*i*_)]. The graph’s global attribute **u** is a tensor with dimensions 3 × *h* × *w*. The first two channels correspond to the map $M\in R^{2\times h\times w}$ , which represents occupancy by tall and short barriers in its first and second channels, respectively. The last channel of **u** corresponds to the latent variable **z**.

One processing stream of the generator reasons about the graph G focusing on the *spatial–orientational arrangement* of the interactants (i.e., the information in the node features) using a GNN that operates in the same spirit as deep sets (Qi et al., 2017; Zaheer et al., 2017)—similar to the “context transform” proposed by Swofford et al. (2020). Another parallel stream processes the graph focusing on *proxemics*, that is, how interactants use space in relation to the environment (Hall, 1966). This stream is a GNN that uses 2D convolutional layers to reason about two types of spatial relationships: the shape of the formation based on the location of interactants (encoded in the vertices of G ) and the location of nearby objects relative to the group (based on the map in the graph’s global attribute).

The generator concatenates the vector representations that result from the two computation streams and then transforms the data through a two-layer perceptron (with ReLU transformations) and one additional linear layer. This results in a 4D output vector, whose first two elements correspond to the position of the output pose $\bar{p}$ . The last two elements are the cosine and sine of the robot’s orientation, which are constrained to lie in (−1, 1) through a final hyperbolic tangent transformation applied to these elements.

The next sections explain how the parallel streams of the generator network are implemented. More implementation details are provided in the Supplementary Material.

##### 4.3.1.1 Spatial–orientational GNN

**FIGURE 4:**
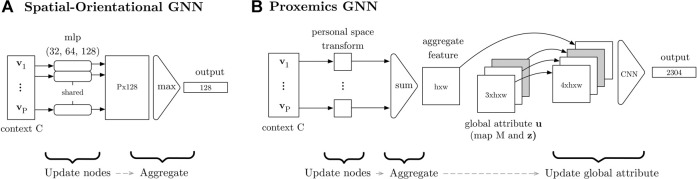
Graph neural networks used in the generator. The “mlp” component in **(A)** is a multi-layer perceptron and “CNN” in **(B)** is a convolutional neural network.

Figure 4A illustrates the architecture of the spatial–orientational component of the generator. The network is a GN block that aggregates position and orientation information from the group: $u_{1}^{\prime }=\rho_{1}^{v\to u}\left({\left\{\phi_{1}^{v}\left(v_{i}\right)\right\}}_{i=1:P}\right)$ , where the update function $\phi_{1}^{v}$ corresponds to a multilayer perceptron (with ReLU activations) applied to the vertex features **v**_*i*_, and the aggregate function $\rho_{1}^{v\to u}$ is max pooling. Comparing these operations with Eqs. 1–3, this GN block can be thought of as having a trivial *ϕ* ^*u*^ function in Eq. 3 that simply returns the aggregate feature for the nodes.

##### 4.3.1.2 Proxemics GNN

Figure 4B depicts the generator’s proxemics component, which is also a GN block. First, the GN block updates the node features by creating a 2D tensor $v_{i}^{\prime }=\phi_{2}^{v}\left(v_{i}\right)\in R^{h\times w}$ that represents the personal space of the interactant *i* using a simple Gaussian blob. That is, $v_{i}^{\prime }$ is a matrix of the same width and height as the map *M*, where each cell corresponds to a physical location in the world and has a value equal to the probability density of a normal distribution centered at the location of the interactant [*x*_*i*_ *y*_*i*_]. Second, the GN block aggregates the updated node features ${\bar{v}}^{\prime }=\sum_{i=1:P}v_{i}^{\prime }$ using element-wise summation. Third, the global attribute is updated by concatenating **u** (with the map and latent variable **z**) with the aggregated personal space representation ${\bar{v}}^{\prime }$ , resulting in a tensor in $R^{4\times h\times w}$ . The latter tensor is then processed by a three-layered convolutional neural network with ReLU activation, and the result is finally flattened into a vector representation $u_{2}^{\prime }$ for this stream. Note that the node update and aggregate functions used by this GNN lead to a representation similar to the personal space loss used for the Geometric approach (and illustrated in Figure 2C). However, the network is not told explicitly how to reason about this data; instead, it needs to figure this out through the adversarial training regimen implemented with the critic.

#### 4.3.2 The Critic Network

We implement the critic in a similar fashion to the generator, with two data processing streams. The main difference is that instead of getting an input graph whose global attribute contains a latent variable **z**, the global graph attribute **u** = *M* in this case. Also, the critic gets an additional input pose **p**, which may come from the dataset D or from the output of the generator. This pose is processed in a third parallel stream, as illustrated in Figure 3B, using a two-layer perceptron with ReLU activations. The three-vector-representations output by the two GNNs and the pose streams are concatenated and finally projected into a scalar value. The Supplementary Material provides more details on the GNNs and this last transformation.

### 4.4 Generating a Distribution of Poses

Both the geometric and WGAN approach described previously can be used to generate a nonparametric distribution of poses for conversational group formations. This is useful in two ways: (1) it can help identify multiple poses that may be suitable for a given conversational group, and (2) it can help overcome predictions that are not optimal, perhaps because of local minima. The latter is particularly important for the Geometric approach because its output is subject to the initial location provided to its optimization routine. Also, computing a distribution can be useful for the WGAN because its generator is not guaranteed to output an ideal pose given an arbitrary input latent vector **z**. Indeed, the neural network is trained to model the distribution of the real data, not a single pose.

Generating a distribution of poses with both approaches is trivial. For the Geometric approach, we can start its optimization step from different initial locations around the context *C*, predicting various locations for the agent. Then, we can compute suitable orientations for each of the locations as explained in Section 4.2. For the WGAN, we simply need to run the generator multiple times using different latent variables as input. Once a nonparametric distribution of poses is computed, we can choose a single pose as output if desired. For example, in our evaluation in Sections 5–6, we do this by searching for a mode of the predicted locations using the mean shift algorithm (Comaniciu and Meer, 2002) and then simply outputting the pose in the distribution that is closest to this mode. We also tried more involved approaches such as computing a mode for the angle of the pose as well using a von Mises kernel density estimator (Fisher, 1995). However, the former approach gave similar or better performance in practice and reduced the number of hyperparameters that we needed to consider in our implementation, facilitating future reproducibility.

## 5 Evaluation on the Cocktail Party Dataset

This section first evaluates the proposed approaches quantitatively with respect to different metrics that describe key properties of F-Formations and desired output poses. Then, we discuss the results qualitatively.

### 5.1 Datasets

We used the Cocktail Party dataset (Zen et al., 2010) to evaluate the proposed approaches. The dataset consists of approximately 30 min of interaction data. It includes 320 frames with conversational group annotations and pose information for six individuals who took part in a Cocktail Party event, as shown in Figure 1A. While the original dataset provides head orientation for each of the individuals based on automatic tracking methods, our evaluation used manually annotated body orientations (Vázquez et al., 2015b) as *θ* for the pose of interactants. Reasoning about body orientation instead of head orientation preserves consistency with the theory of F-Formations (Kendon, 1990). In addition to this data, we manually created an environment map for the Cocktail Party scene with labels for “free space,” space occupied by “tall objects” (through which social interactions are unlikely), and space occupied by “short objects” (like the table in the room). Areas outside of the Cocktail Party room were labeled as having “unknown” occupancy in the map and were treated as occupied space in practice.

We split the group annotations from the Cocktail Party dataset into two sets: training (80%) and testing (20%). The test set included 31 frames with group annotations at the beginning of the Cocktail Party sequence, 31 frames in the middle, and 31 more at the end; the training set was composed of the other frames with group annotations.^1^ The latter groups were then used to create a dataset $D_{\text{train}}^{\text{CP}}=\left\{<C,M,p>\right\}$ of 1,394 examples with corresponding contexts *C*, map *M*, and example ground truth pose **p** for a robot. The map for these examples had 24 × 24 cells and a resolution of 0.25 m per cell. They were a cropped section (generated with subpixel accuracy) of the full environment layout, covering an area of approximately 3-m radius around the context *C*. The ground ruth pose in the examples corresponded to the position and orientation of one member of the group who was excluded from the context. Using the test groups, we created a similar dataset $D_{\text{test}}^{\text{CP}}$ for evaluating the proposed models, where $|D_{\text{test}}^{\text{CP}}|=347$ .

**FIGURE 5:**
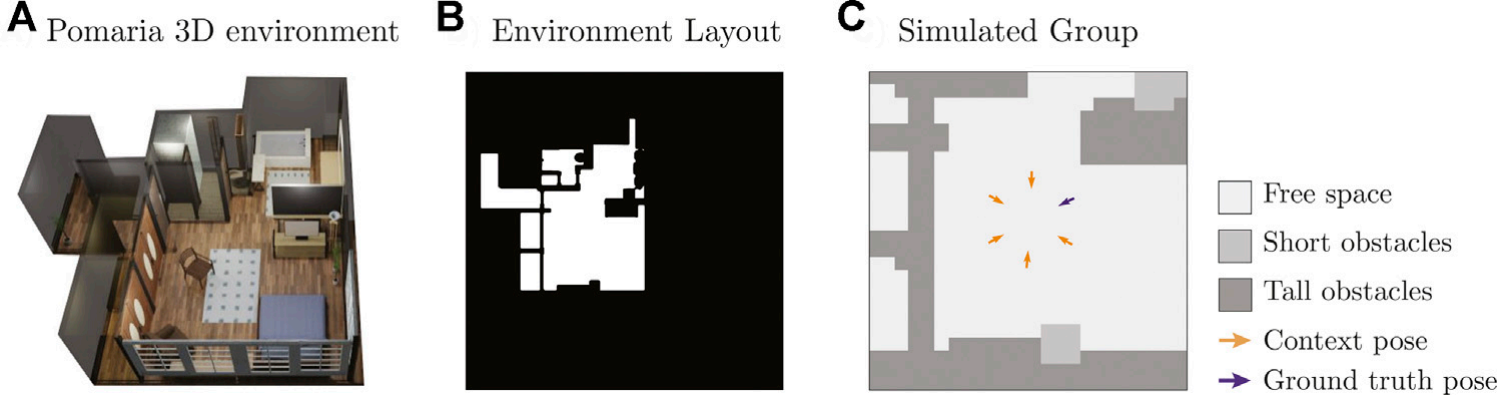
**(A)** Example 3D environment from iGibson (Shen et al., 2020), **(B)** environment layout generated from the 3D environment, and **(C)** simulated sample on a cropped section of the layout.

We also created a dataset of simulated F-Formations using 15 environment layouts from the iGibson simulation environment (Shen et al., 2020). For each environment, we first created a 2D layout intersecting the 3D geometry of the world with planes parallel to the ground, as illustrated in Figures 5A, B. Using the layout, we then manually created an environment map with the same labels and resolution of the Cocktail Party environment map and automatically generated circular groups with two to six people in free areas of the environment following a simple rule-based procedure. This resulted in 34,405 simulated examples, each with a corresponding environment map, context and example ground truth pose for the robot. Figure 5C shows one sample from this dataset.

Upon preliminary testing of the data-driven method, we realized that the WGAN significantly benefited from many diverse examples. Thus, we further augmented the dataset of simulated groups by warping the data using a small amount of horizontal and vertical stretch as well as random rotations. This resulted in an expanded dataset of 60,365 simulated examples in total, which we used to train some variations of the data-driven model in this evaluation. The Supplementary Material provides more details about the data generation process used to create simulated F-Formations in iGibson environments.

### 5.2 Pose Generation Methods

The present evaluation considered variations of the proposed methods and a recent baseline for robot pose generation in F-Formations, which does not use information about the surrounding physical environment. To the best of our knowledge, no prior work has considered variability in the environment of F-Formations when generating suitable poses for an interactant. All methods were implemented in Python.

Baseline method: We implemented the pose generation method by Yang et al. (2017). As with our model-based approach, this method seeks a circular spatial pattern but without explicitly accounting for environment characteristics. Instead, the existing social context alone determines the generated pose, which is computed as follows: First, any pair of individuals in the context is used to define a mutual circular region. Second, all pairwise centers are averaged to compute the center coordinate of the o-space, to which the new member faces. The minimum and maximum distances of individuals to the common center demarcate the p-space of the group, the annular zone that interacting peers occupy (as in Figure 1B). Finally, bisecting the largest gap between adjacent neighbors identifies the new member’s position within the group.

Geometric methods: We evaluated the Geometric approach proposed in Section 4.2 considering two cases. In one case, the method generates a single pose using an initial location for its optimization step that is within a 3 × 3 m region around the center of the given context *C*. In the other case, we run the method multiple times to model a distribution of poses and then use mean shift to choose an output pose (as described in Section 4.4). In the latter case, we initialize the method with 36 different initial values for its optimization, which are sampled uniformly in the same 3 × 3 m region considered in the former case. For both variations, we set the loss parameters *σ* = 0.21, *λ*_*p*_ = 1.25, *λ*_*c*_ = 0.2 and *λ*_*f*_ = 0.5 based on initial results on $D_{\text{train}}^{\text{CP}}$ .

Data-driven methods: We considered three variations of the proposed WGAN. First, we considered a model trained on the simulated dataset (with a small amount of angular noise applied to the orientation of the context poses to make the group arrangements more varied). Second, we evaluated a model trained on the Cocktail Party train data only (with 10*%* used for validation). Third, we considered a model trained like the first one and then fine-tuned on the Cocktail Party train data. In addition, we considered generating one sample pose from the generator, as well as generating a distribution of 36 poses from which we output a solution guided by mean shift (as in Section 4.4).

We implemented the WGAN using the PyTorch library and trained models using an NVidia GeForce RTX 2080 Ti GPU. More specifically, we used the Adam optimizer with a learning rate of 0.00002, a batch size of 32, and a weight of 10 for the WGAN gradient penalty (Gulrajani et al., 2017). During gradient descent, we weighted the training samples based on the relative distribution of group sizes in the dataset and updated the critic five times for every generator update. We trained models for at least 600 epochs and chose the best training weights through a combination of manual inspection of the generated samples and quantitative metrics on the Cocktail Party validation data.

The Supplementary Material provides more implementation details for the WGAN. Also, it describes results for several other variations of the WGAN that we explored in this work, but that resulted in no major improvement. For example, we considered a model that only had information about free space, instead of multiple map labels.

### 5.3 Quantitative Metrics

We considered a range of metrics that describe F-Formations and social norms in regard to spatial behavior:

– Deviations from fitted circle or ellipse (Circ. Fit). We measure the perpendicular distance from a generated pose to a circle or ellipse that has been fitted to the context *C*. The circle or ellipse is fitted following the same considerations described in Section 4.2 for the proposed Geometric approach.
– Individual is not on free space (Not Free). We compute how often the location of a generated pose falls within a nonfree cell in the environment map *M*. The values for this metric ranged in [0, 1] because of subpixel cropping of the maps.
– Violations to personal space (Per. Space). We compute the number of cases in which the distance between the generated pose $\bar{p}=<x,\theta >$ and the pose of another member of the group < **x**_*j*_, *θ*_*j*_ > is less than a personal space threshold, ‖**x** − **x**_*j*_‖ < *δ*. We use a threshold of *δ* = 0.68 m based on real-world data of interpersonal distances in Italy (Sorokowska et al., 2017), because the Cocktail Party data were originally captured in that country.
– Violations to intimate space (Int. Space). Similar to personal space, we compute the number of cases in which the distance between the generated pose and another group member *j* is less than an intimate space threshold, ‖**x** − **p**_*j*_‖ < *ρ*. We use *ρ* = 0.42 m based on Sorokowska et al. (2017).
– Distance to group’s o-space center (Center Dist.). Let **x**_*i*_ and *θ*_*i*_ be the location and body orientation of a social agent (human or robot) in a conversational group. Prior work, such as those of Cristani et al. (2011) and Setti et al. (2013, 2015), has proposed to compute the o-space center of an F-Formation as follows:

$$\bar{o}=\frac{1}{P}\overset{P}{\underset{i=1}{\sum }}o_{i}=\frac{1}{P}\overset{P}{\underset{i=1}{\sum }}\left(x_{i}+d\left[\begin{array}{c} \cos\left(\theta_{i}\right)\\ \sin\left(\theta_{i}\right) \end{array}\right]\right)$$ (6)

where *P* is the number of interactants in the group, and **o**_*i*_ is a proposed o-space center for member *i*. Thus, we measure alignment with an ideal F-Formation model as the average distance between the group’s o-space center and the o-space center proposals for individual members: $CenterDist=\frac{1}{P}\sum_{i=1}^{P}\|\bar{o}-o_{i}\|$ . For the parameter *d*, needed to compute $\bar{o}$ in Eq. 6, we use *d* = 0.72 as it minimizes $\sum_{g=1}^{K}\sum_{i=1}^{P^{g}}{\left\|{\bar{o}}_{g}-o_{i}\right\|}^{2}$ , considering all *K* ground-truth groups for the Cocktail Party dataset (see Vázquez (2017) for the derivation).

– Individual occludes another interactant (Occ. Other). Ideally, the generated poses should not be in front of other interactants, as this would prevent them from having direct access to the o-space and exclude them from the group. To identify these situations, we check if the generated pose is in between another interactant in the group and the o-space center $\bar{o}$ . The center $\bar{o}$ is computed as in Eq. 6 while excluding the generated pose, because a bad prediction could skew significantly $\bar{o}$ .
– Individual is behind another interactant (Is Occ.). This metric is similar to the prior metric, but we invert the roles of the generated pose and an interactant’s pose for which we compute the occlusion.

The first three metrics correspond to each of the losses considered by the Geometric approach and thus serve to validate that the method was working as expected. In addition, these metrics are useful to evaluate whether the data-driven method behaved in a similar manner. The occlusion metrics are inspired by the visibility constraints from Vázquez et al. (2015b) and Setti et al. (2015), and the personal and intimate space metrics signal potential violations to social norms. Lastly, the Center Dist. metric serves to evaluate the combined effect of position and orientation prediction. The metrics are inspired by ideal models of F-Formations, but real-world data may not perfectly satisfy all assumptions set forth by the metrics. Thus, we report values for ground truth test data in our results as a reference for comparison.

### 5.4 Quantitative Results

Table 1 presents the results on the Cocktail Party test set. As a reference, the first row shows the values for the metrics using ground truth poses from the test set (which were removed to create the context *C* input to the pose generation methods shown in Table 1).

**TABLE 1:** Results on the Cocktail Party test set.

	Method	Circ. Fit	Not Free	Per. Space	Int. Space	Center Dist.	Occ. Other	Is Occ.
1	Ground Truth	0.35 ± 0.24	0.01 ± 0.09	0.48 ± 0.56	0.00 ± 0.00	0.27 ± 0.10	0.00 ± 0.00	0.00 ± 0.00
2	Yang	3.02 ± 21.51	0.26 ± 0.43	0.28 ± 0.62	0.00 ± 0.05	1.20 ± 9.44	0.00 ± 0.00	0.02 ± 0.14
3	Geometric	0.33 ± 0.29	0.00 ± 0.05	0.01 ± 0.13	0.00 ± 0.00	0.30 ± 0.24	0.01 ± 0.27	0.09 ± 0.28
4	WGAN (iG)	0.33 ± 0.29	0.07 ± 0.23	0.38 ± 0.61	0.11 ± 0.33	0.45 ± 0.15	0.03 ± 0.17	0.01 ± 0.12
5	WGAN (CP)	0.28 ± 0.23	0.02 ± 0.13	0.73 ± 0.71	0.31 ± 0.49	0.46 ± 0.12	0.10 ± 0.40	0.06 ± 0.23
6	WGAN (iG, CP)	0.31 ± 0.23	0.03 ± 0.16	0.68 ± 0.64	0.22 ± 0.41	0.45 ± 0.11	0.12 ± 0.32	0.01 ± 0.12
7	Geometric^*^	0.29 ± 0.28	0.00 ± 0.05	0.01 ± 0.13	0.00 ± 0.00	0.30 ± 0.24	0.00 ± 0.05	0.07 ± 0.26
8	WGAN* (iG)	0.33 ± 0.28	0.07 ± 0.23	0.36 ± 0.59	0.10 ± 0.29	0.45 ± 0.15	0.03 ± 0.18	0.01 ± 0.09
9	WGAN* (CP)	0.29 ± 0.23	0.02 ± 0.13	0.72 ± 0.68	0.32 ± 0.49	0.46 ± 0.12	0.12 ± 0.40	0.04 ± 0.20
10	WGAN* (iG, CP)	0.31 ± 0.22	0.03 ± 0.15	0.66 ± 0.65	0.21 ± 0.41	0.45 ± 0.11	0.11 ± 0.32	0.02 ± 0.13

Each row shows µ ± σ for each of the metrics described in Section 5.3 (lower is better). Models without * output a single pose, whereas those with * output a distribution of 36 poses from which we chose a single pose (guided by the mode of the distribution) as final output. “(iG)” models were trained on simulated data using iGibson environment maps, “(CP)” indicates training with Cocktail Party train data, and “(iG,CP)” corresponds to pretraining with simulated data and then fine-tuning on Cocktail Party train data. The best results (for which there are no significant differences) are highlighted in gray per column—see the text for statistical analyses.

Unless noted otherwise, we analyzed the results for the quantitative metrics using restricted maximum likelihood (REML) analyses considering method (10 levels, each one corresponding to a row of Table 1) as main effect and Example ID from the Cocktail Party test set as random effect. The results for the Circ. Fit metric indicated a significant effect of method (*F*[9, 3114] = 5.50, *p* < 0.0001). A Tukey honestly significant difference (HSD) *post hoc* test showed that the baseline method by Yang et al. (2017) led to significantly higher Circ. Fit values than the other methods. The baseline performed poorly because its o-space representation is the average of all circles fitted to pairs of group members. Thus, a single pair can heavily bias the position of the generated interactant. For example, we often observed this bias when the difference between the orientations of a pair of individuals in the context was small, which resulted in a circle with a disproportionately long radius. There were no other significant pairwise differences for the Circ. Fit results, suggesting that the proposed methods were able to effectively capture the circularity of F-Formations.

An REML analysis on the Not Free metric indicated that there were significant differences by Method (*F*[9, 3114] = 64.72, *p* < 0.0001). The *post hoc* test showed that the baseline method by Yang et al. (2017) resulted in significantly more poses generated in occupied cells of the environment map than all other methods. This was expected because the baseline did not consider the environment map in its calculations. The only other pairwise differences for the Not Free metric were the results for rows 4 and 8 in Table 1, which were low but significantly higher than the results for rows 1, 3, 5, 7, and 9. As a reference, rows 4 and 8 corresponding to the WGAN trained on iGibson-simulated data led to 24/347 and 22/347 examples for which the Not Free metric was greater than 0.5. Meanwhile, the ground truth values had 3/347 instances in this category, and the Geometric approach led to only one such case in the Cocktail Party test set.

We also found significant differences for violations to personal space (*p* < 0.0001) and intimate space (*p* < 0.0001) using REML analyses, as well as using Poisson generalized mixed linear models with a log link function. In terms of Per. Space, a Tukey HSD *post hoc* test showed that the Geometric approach (rows 3 and 7 in Table 1) led to significantly lower number of personal space violations than all other methods, followed by the Yang baseline (row 2) and the WGAN trained on simulated data using iGibson environments (rows 4 and 8). Also, the WGAN trained or fine-tuned on Cocktail Party train data led to significantly higher violations to Per. Space than all other methods. In terms of Int. Space, best results were obtained with the Ground Truth poses (row 1), the Yang baseline (row 2), and the Geometric approaches (rows 3 and 7). These methods had significantly fewer intimate space violations than all other methods. Further, the WGAN trained on simulated data (rows 4 and 8) was significantly better in terms of Int. Space than the other WGAN variations (rows 5, 6, 9, and 10). The WGAN fine-tuned on Cocktail Party train data (rows 6 and 10) was also significantly better than the WGAN trained on these data only (rows 5 and 9).

The results for the Center Dist. metric were similar to the Circ. Fit metric: an REML analysis showed significant differences per Method (*F*[9, 3114] = 2.75, *p* = 0.003), and the *post hoc* test showed that the Yang baseline had significantly worse Center Dist. results than all the other methods.

The values for the occlusion metrics were generally low, but there were significant differences across Methods. For Occ. Other (*p* < 0.0001), the methods in rows 1–4, 7, and 8 in Table 1 resulted in poses that led to significantly fewer occlusions than the methods in rows 5, 6, 9, and 10. For the Is Occ. metric (*p* < 0.0001), the Tukey HSD *post hoc* indicated that the Ground Truth results (row 1 in Table 1) were significantly lower than those for the Geometric approach (rows 3 and 7) and the WGAN trained on the Cocktail Party train data (rows 5 and 9). However, there were no significant pairwise differences between the Ground Truth results, the Yang baseline (row 2), the WGAN trained on iGibson data (rows 4 and 8), or the WGAN fine-tuned on Cocktail Party train data (rows 6 and 10).

In summary, the results in Table 1 led to three key takeaways. First, the proposed methods worked better than the baseline in terms of the Circ. Fit, Not Free, and Center Dist. metrics. This showed the value of considering environmental spatial constraints when predicting poses for agents in conversational groups and the superiority of the proposed methods at modeling the shape of F-Formations. Second, training the WGAN on simulated data using iGibson environments turned out to be as good as or better than training on realistic Cocktail Party data only, except for the Not Free metric for which the simulated data led to slightly worse results. We attribute this result to the fact that generative adversarial models are data-hungry, and the Cocktail Party train set had only 1,394 examples (approximately 2% of the simulated dataset). Effective fine-tuning of the WGAN model on the small Cocktail Party train set proved difficult. Third, computing a distribution of poses led to slight improvements in some cases compared to predicting a single pose directly. For instance, the distribution helped slightly the WGAN model in terms of personal space violations and the Geometric approach in terms of occlusions.

### 5.5 Qualitative Results

**FIGURE 6:**
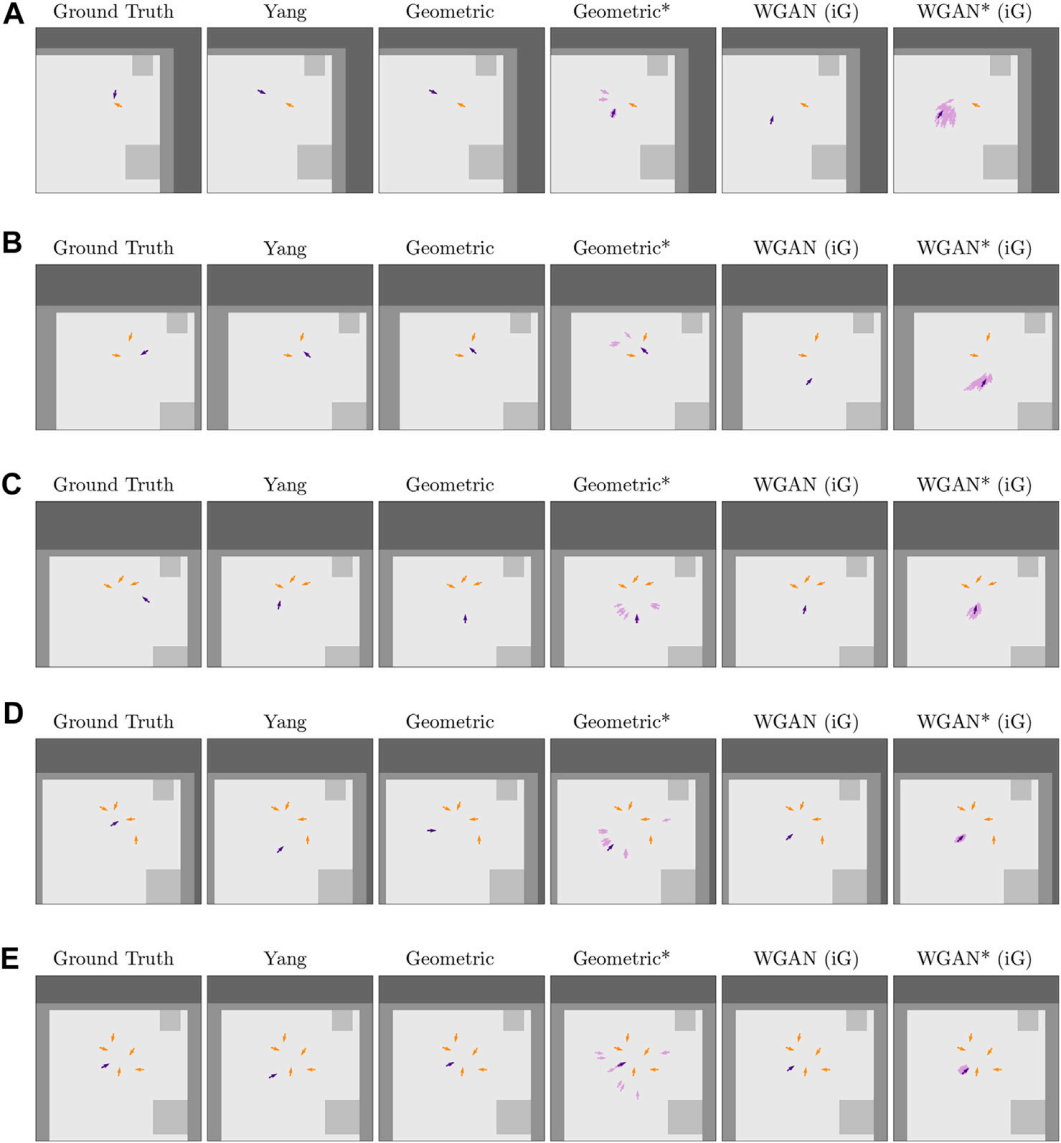
Successful predictions for several methods (one per column) on five different problems (rows) from the Cocktail Party test set. The orange arrows correspond to the poses in the context *C*, and the purple arrows are predicted poses, except for the Ground Truth column in which the purple arrows correspond to a true pose by a group member. Note that the darker purple arrows are the final output by each method, and the lighter ones are additional predictions by the methods that computed a distribution of poses. The colors of the environment map are the same as in Figure 5C: “free space” is light gray, “short obstacles” is medium-intensity gray, and “tall obstacles” is darker gray. In addition, these plots show one more label for “unknown” occupancy (darkest gray color). The latter label was a result of cropping the full environment map around context poses next to the edge of the map. When computing results, “unknown” occupancy was considered as nonfree space by the Geometric approach and was aggregated with tall obstacles for the WGAN. This Figure is best viewed in color.

We further analyzed the results from Section 5.4 qualitatively for the baseline by Yang et al. (2017), the Geometric approach and the WGAN (trained on simulated data). Figures 6A–E shows example results by these methods on different group sizes. The columns are identified with the same naming convention as Table 1, where ^*^ in the Figure corresponds to methods that internally predicted a distribution of 36 poses.

In comparison to the baseline (Yang column), the proposed Geometric approach resulted in similar predictions when the context had one or two poses (Figures 6A,B). However, for bigger groups, the Geometric approach tended to model circular spatial arrangements more consistently than the baseline, resulting in poses that were better positioned or oriented with respect to the context.

In regard to the methods that computed pose distributions, Figure 6 shows that these distributions captured different viable solutions to the pose generation problem. Interestingly, while the Geometric^*^ approach tended to lead to more multimodal distributions than the WGAN* (iG), the data-driven method led to fewer occluded poses in these distributions. Occlusions were a problem for the Geometric approach due to local minima in its optimization step, but by predicting multiple poses, this problem was alleviated.

**FIGURE 7:**
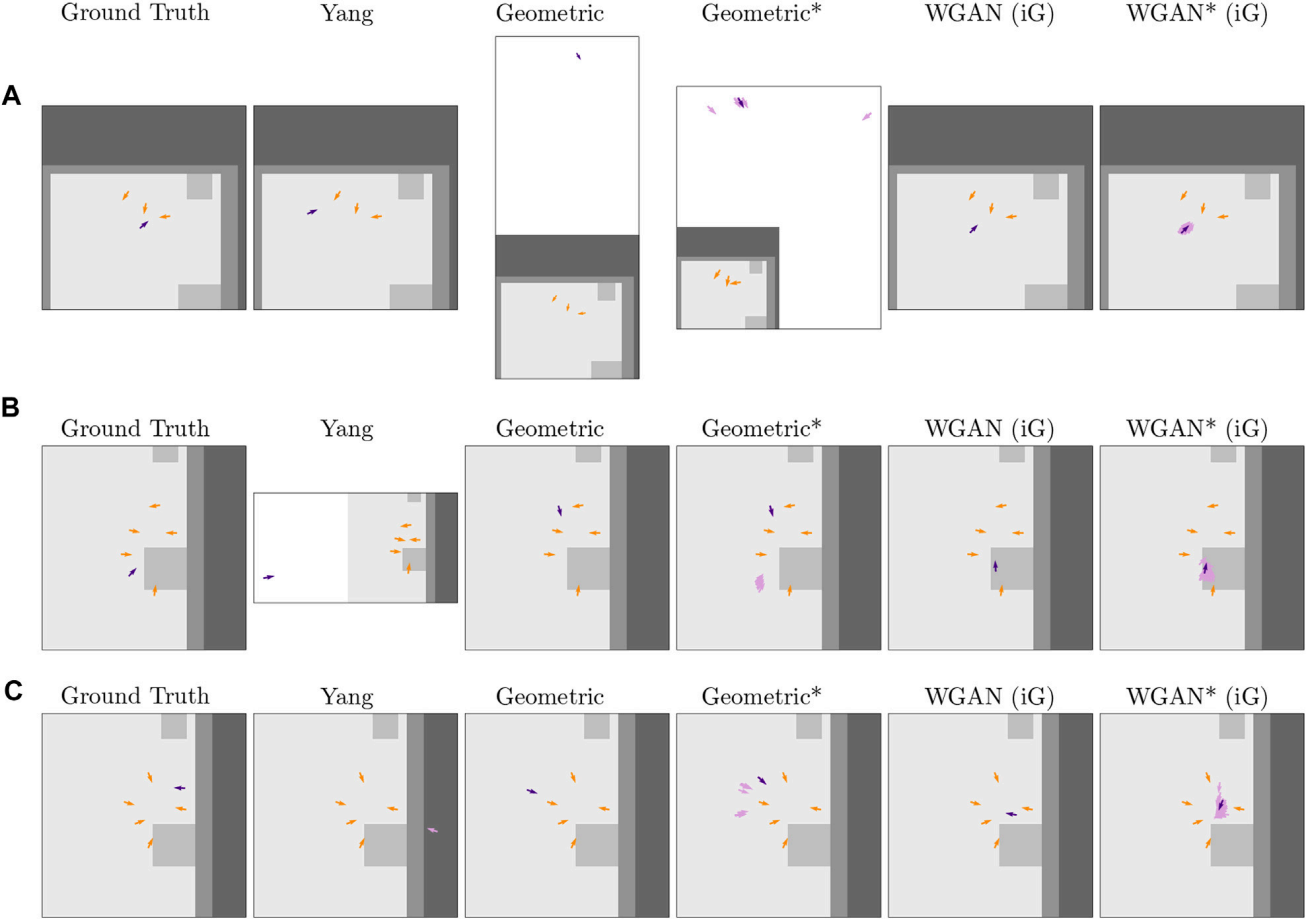
Difficult predictions on three different problems (rows) from the Cocktail Party test set. As in Figure 6, the orange arrows correspond to the poses in the context *C*, the purple arrows are Ground Truth or predicted poses, and the lighter gray color in the maps corresponds to free space. White areas within a plot correspond to regions of the space out of the cropped map (considered as “unknown” occupancy by the proposed methods). This Figure is best viewed in color.

Figure 7 shows more difficult prediction problems, where the context poses are distributed in less circular form or are closer to physical obstacles. These cases led to poor o-space modeling for both the baseline and the Geometric approach. In particular, in Figure 7A, the Geometric approach fit a circular shape to the context that had a disproportionately big radius and was oriented in the wrong direction. In Figure 7B, the baseline by Yang et al. (2017) had trouble with pairs of poses in the context being oriented very similar to one another, which led to a generated pose that was very far away from the group. Also, in Figure 7C, the baseline output a generated pose in nonfree space.

In terms of the WGAN, Figure 7B shows that the WGAN had more trouble avoiding short obstacles than the other methods. Furthermore, Figure 7C shows that another failure for the WGAN was to place poses toward the center of a group. Predicting a distribution of poses in this case was useful in comparison to generating a single pose, as the distribution comprised poses in more appropriate positions relative to the context.

Despite the challenges encountered in some cases by the proposed approaches, they generally performed better than the baseline by Yang et al. (2017) both in terms of considering environmental constraints and dealing with the variability inherent in human spatial behavior. However, it was hard to evaluate the methods holistically: we did not know of a good way to combine the quantitative metrics considered in this section into a single success measure. Thus, to complement these results, we conducted a complementary, human-driven evaluation of the proposed approaches. This evaluation is presented in the following section.

## 6 Human Evaluation

We evaluated generated poses by the proposed geometric and data-driven approaches from a human perspective. For this evaluation, we chose a diverse set of the groups from the Cocktail Party test data. We then removed one human member of the groups, as in the prior evaluations, and computed the pose for a robot to be part of the interaction with the remaining members. The resulting spatial arrangement was rendered in a virtual scene similar to the environment of the Cocktail Party dataset. Human participants then gave us their opinion of the pose of the robot relative to the virtual humans rendered in the scenes.

This experiment followed a similar protocol to Connolly et al. (2021). The main difference is that our focus was not on evaluating the effect of different robot embodiments on human perception of conversational groups; instead, we wanted to compare the two proposed methods for pose generation. For this reason, we focused on using a single robot embodiment for this study. The selected robot was a humanoid Pepper robot, which has an easily discernible body orientation and head. Its dimensions are similar to those of a young person.

### 6.1 Participants

We used Prolific to recruit a total of 60 participants (32 females and 28 males) for this human evaluation. The participants resided in the United States, were fluent or native English speakers, had normal or corrected-to-normal vision, and had an average age of 32.15 years (standard deviation [STD] = 12.57). They indicated sometimes playing video games (mean [M] = 4.32, STD = 2.14) and rarely interacting or working with a robot (M = 2.23, STD = 1.59) on 7-point responding formats (1 being the lowest rating and 7 being highest).

### 6.2 Experiment Design

We controlled for three main variables in this evaluation:

Method (two levels). We compared the Geometric approach (Section 4.2) with the WGAN approach (Section 4.3). Both methods computed a distribution of 36 samples from which we chose a single output pose by searching for a mode across predicted locations (as explained in Section 4.4). For both methods, we used the best hyperparameters found in Section 5. For the WGAN, in particular, we chose the model that was trained on simulated iGibson data (row 8 of Table 1) because, except for the Not Free metric, it led to better or similar performance than alternatives.

Context (10 levels). We considered 10 contexts (i.e., interactants’ poses input to the methods) for each of the Group Sizes mentioned below. The contexts were chosen to try to maximize the diversity of scenarios considered in the evaluation and without looking at how well the proposed methods performed on them.

Group Size (five levels). We considered group sizes of two to six interactants (including the robot). This means that the proposed methods had as input a Context with one to five interactants.

**FIGURE 8:**
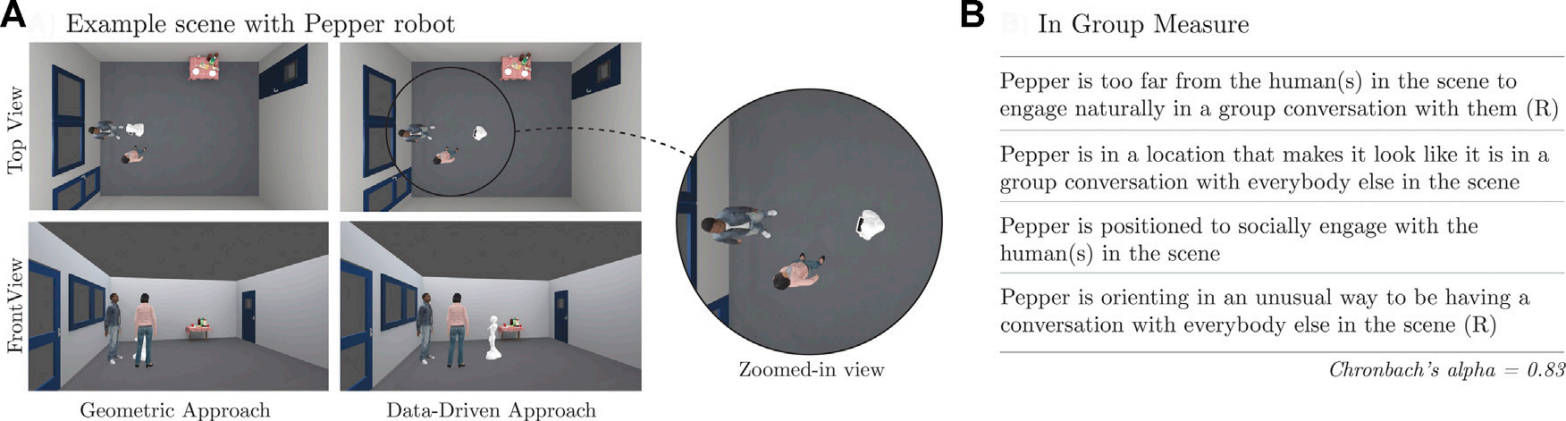
Example scene used for the human evaluation **(A)** and statements rated by the participants about the robot’s pose **(B)**. (R) indicates that the ratings were reversed before computing the “In Group” measure.

The study was run with a mixed design using a Qualtrics online survey. The participants provided their opinion of the pose of the robot for a single Group Size (between participants) in renderings generated for all Context/Method combinations (within participants). In particular, for each combination of Context and Method, we generated two renderings that depicted the resulting interaction.^2^ One rendering corresponded to a top-down view of the group, and the other was a frontal view so that the participants could easily perceive the robot’s spatial positioning relative to the other interactants (as shown in Figure 8A).

The participants were randomly assigned to each Group Size category, resulting in all categories having at least four males or females. Renderings made for Group Sizes of three, four, and six interactants were evaluated by 12 participants each, whereas the renderings for Group Sizes of two and five interactants were evaluated by 13 and 11 participants, respectively.

### 6.3 Measures

The participants provided feedback about the pose of the Pepper robot on each scene shown in their survey, each of which corresponded to a given combination of Context and Method. In particular, the survey first asked them to visually identify the Pepper robot in the rendered scene. Then, it asked them to rate four statements about the robot’s pose relative to the virtual humans. Example images can be seen in Figure 8, along with the statements that the participants had to rate using a 7-point Likert responding format from “strongly disagree” (1) to “strongly agree” (7). Following Connolly et al. (2021), we reversed the scores for negative statements and computed the correlation between them, obtaining moderate positive pairwise correlations (see Supplementary Table S8 in the supplementary material). Cronbach *α* for these ratings was 0.83, above the nominal 0.7 threshold. Thus, we grouped responses into an “In Group” measure.

### 6.4 Procedure

Upon starting the survey, the participants completed a consent form to take part in the evaluation and provided basic demographics data (as described in Section 6.1). Then, the survey showed renderings of two practice scenes and asked the participants to rate the pose of the robot in them using the In Group statements from Figure 8B. The practice scenes depicted different Contexts than those used for the evaluation to avoid biasing participant’s opinion. In one practice scene, the pose of the robot corresponded to the ground truth pose for the individual that it replaced in the Cocktail Party dataset. In the other practice scene, the robot’s pose was generated by taking the ground truth pose and then reorienting the robot opposite to its group. These examples served to familiarize participants with the robot and the In Group statements used to rate its pose.

After the two practice scenes, the survey showed the real evaluation scenes. For each scene, the survey asked the participants to evaluate the pose of the Pepper robot using the In Group statements. Note that the survey for the participants who provided feedback for groups of size 4 included only 19 evaluation scenes because the Geometric approach led to positioning the robot outside of the Cocktail Party environment in one case, which we removed from our evaluation. For all other group sizes, the survey included 20 evaluation scenes as originally planned (10 contexts × 2 methods). The order of the evaluation scenes was randomized for all the participants. That is, the renderings by Method and Context were randomly interspersed with one another within a participant’s survey to avoid potential ordering effects.

After rating all the scenes, the participants provided their opinion about how hard it was to complete the survey. We used these responses in pilots to improve the protocol design. The survey typically took approximately 12 min to complete, for which the participants were paid US $2.4. This protocol was approved by our local Institutional Review Board.

The Supplementary Material provides more details on the specific design of the online survey and shows all the renderings used in this evaluation.

## 6.5 Results

We conducted an REML analysis on the In Group measure. In this analysis, we considered Method, Group Size, and their interaction as main effects, and Context and Participant ID as random effects. We found significant effects for Group Size (*F*[4, 55.19] = 3.24, *p* = 0.019). A Tukey HSD *post hoc* test suggested that the In Group ratings were significantly lower on groups of size 4 (M = 4.44, STD = 1.67, N = 240) than on groups of size 6 (M = 5.27, STD = 1.65, N = 240). No other significant differences were obtained by Group Size. The ratings for groups of size 2, 3, and 5 were M = 4.78 (STD = 1.37; N = 260), M = 5.14 (STD = 1.56; N = 240), and M = 4.91 (STD = 1.58; N = 220), respectively.

The REML analysis indicated that Method had a significant effect on the In Group ratings, *F*[1, 1115] = 10.06 (*p* = 0.002). A Student *t post hoc* test suggested that the data-driven approach (M = 5.01, STD = 1.44, N = 600) led to significantly higher ratings than the Geometric approach (M = 4.81, STD = 1.73, N = 600), although this difference was small (approximately 0.2 points on the 7-point scale).

**FIGURE 9:**
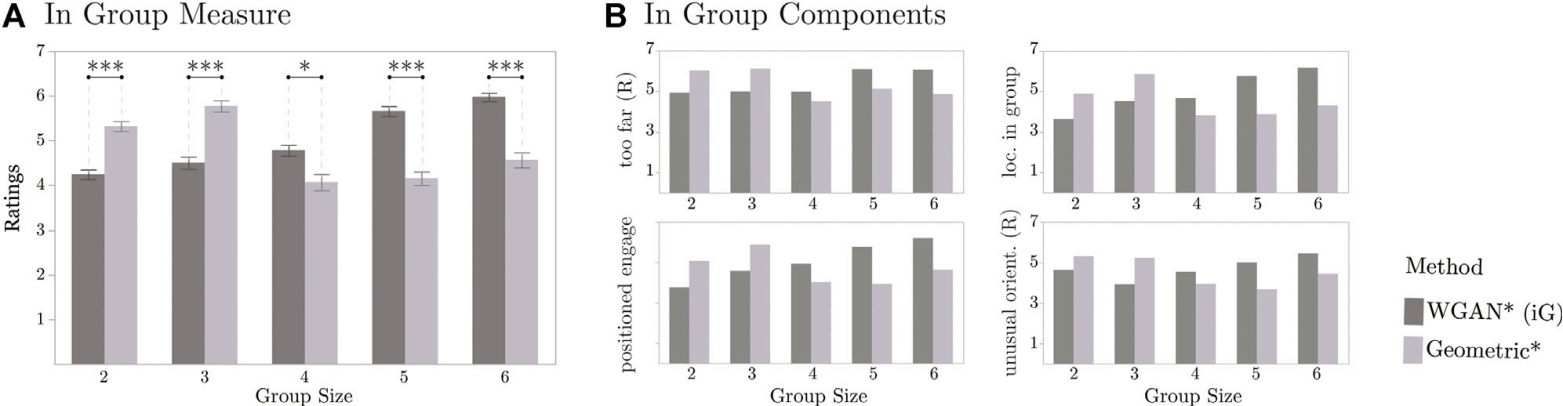
Results from online human evaluation comparing the Geometric and data-driven approach (WGAN). In the left plot, the symbol * indicates *p* < 0.05 and *** indicates *p* < 0.001, and error bars correspond to standard. error. The right plots only show averages to illustrate the similarity among ratings.

There was also a significant interaction effect of Method and Group Size on the In Group measure, *F*[4, 1114] = 61.43 (*p* < 0.0001). Interestingly, a Tukey HSD *post hoc* test indicated that the In Group ratings were significantly higher for the WGAN than for the Geometric approach on Group Sizes 4, 5, and 6. However, the Geometric approach led to significantly higher ratings than the data-driven method for the other Group Sizes, as illustrated in Figure 9A. This difference in performance by Group Sizes was observed on each of the individual components of the In Group measure, as shown in Figure 9B.

We looked further into the generated renderings to better understand why the methods led to different In Group values per Group Size. We noticed two trends:

1. For a Group Size of 2 and 3, the WGAN tended to place the robot farther away from the context individuals than the Geometric approach. For example, this result can be seen in Figure 8A. Also, additional examples can be found in Supplementary Figures S4 and S5 in the supplementary material. For instance, for groups of size 2 in Supplementary Figure S4, the robot is farther away from the groups with the WGAN than with the Geometric approach in Contexts 2, 3, 5, 6, 7, 9, and 10. Likewise, this effect can be seen in Contexts 1, 3, 5, 6, and 8 for groups of size 3 in Supplementary Figure S5.
2. For Group Sizes bigger than 3, we observed in the renderings that the Geometric approach tended to place the robot more often behind individuals than the WGAN. For example, this can be seen in Contexts 4, 6, 9, and 10 for Group Size 4 in Supplementary Figure S6 in the supplementary material. Likewise, this result can be seen in Contexts 1, 3, 4, 5, 9, and 10 for Group Size 5 in Supplementary Figure S7, and on Contexts 1, 3, 6, 7, 8, and 9 for Group Size 6 in Supplementary Figure S8. This result is a direct consequence of the hyperparameters that we chose for the model-based method. In particular, when looking at preliminary results in the Cocktail Party train dataset (as described in Section 5), we prioritized avoiding violations to personal and intimate space. However, this impaired the capacity of the Geometric approach to find suitable gaps for the robot in spatial arrangements that already had at least three members.

The mixed results for the In Group ratings highlight different properties of the proposed pose generation methods. First, we attribute the lower In Group ratings for the WGAN on Group Sizes 2 and 3 to the method’s reliance on the training data distribution. As mentioned before, we trained the WGAN using simulated iGibson data, based on our earlier results on the Cocktail Party dataset (Section 5). However, these data were generated without special consideration for group size. All the groups were created by simply placing interactants along a circular arrangement in free space; we should have instead created smaller circular arrangements for smaller groups. Second, the difficulties that the Geometric approach had with Group Sizes 4, 5, and 6 speak to how challenging it is to choose suitable hyperparameters for the Geometric approach given all the many factors that matter for the pose generation problem, including proxemics, the shape of F-Formations, occlusions within groups, and the physical environment.

## 7 Generating Conversational Groups

Although we focused our work on predicting a suitable pose for a social agent in a group conversation, the proposed approaches could be reused to create entire conversational groups. These groups are constructed by invoking the proposed generative methods iteratively given a map *M*, the pose of an initial individual < **x**_0_, *θ*_0_ > , and the desired number of group members. After each iteration, the newest generated pose is added to the social context, which the generator subsequently takes as an input.

**FIGURE 10:**
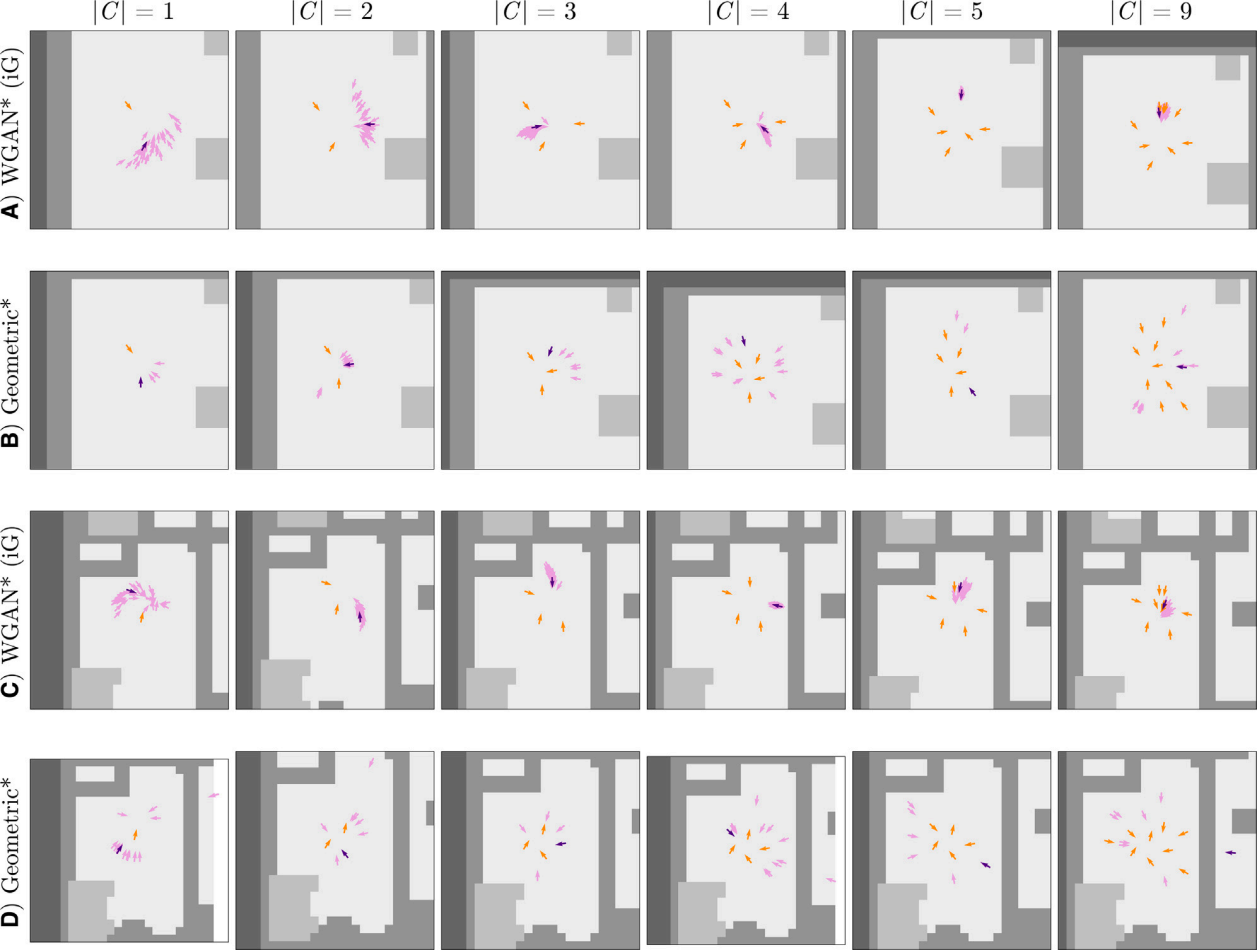
Group generation through iterative invocations of the geometric and data-driven approaches. The plots in rows **(A)** and **(B)** show results in the map from the Cocktail Party dataset. Rows **(C)** and **(D)** demonstrate our two approaches in a map from the iGibson dataset. As in Figure 6, the orange arrows correspond to the poses in the context *C*, the purple arrows are Ground Truth or predicted poses, and the lighter gray color in the maps corresponds to free space. |*C*| indicates the size of the context used to make a prediction per column. This Figure is best viewed in color.

Figure 10 illustrates conversational groups generated by both proposed approaches using the above iterative method on two different environments: one map corresponds to the single room of the Cocktail Party dataset (Figures 10A,B), and the other one is drawn from the iGibson environments (Figures 10C,D). At each iteration of the group generation approach, the final pose output for a new interactant is selected from a distribution of 36 samples computed by the corresponding method. These samples are shown as light purple arrows in Figure 10. The hyperparameters for the Geometric and WGAN methods used in this section are the same parameters used for computing the results in Sections 5 and 6.

In general, the results for the iterative group generation task reflect prior findings. First, the Geometric approach generates poses that better respect personal space, as can be seen in the right-most column of Figure 10. Second, for smaller group sizes, the Geometric approach outputs poses that are more tightly positioned relative to existing group members than the WGAN; however, for bigger group sizes, the WGAN outputs poses in more circular group formations than the Geometric approach. These circular formations are prototypical of real conversational interactions, suggesting that the WGAN better identifies proxemic constraints introduced by additional interactants than the Geometric approach.

From a computational perspective, iterative invocation of the geometric method, without special care for parallelization, requires more time to output a result than the WGAN due to the inherent sequential nature of its optimization step. For example, while the WGAN might take approximately 0.08 s to make a prediction on a consumer-grade MacBook computer, the Geometric approach might take approximately 0.5 s. Owing to this higher runtime cost yet greater stability, the optimization approach may be used to simulate very large groups appropriate for training data-driven models in the future.

Lastly, the results also show limitations of the Geometric approach in reasoning holistically about social scenes. For example, in Figure 10D, the Geometric approach proposes a pose for |*C*| = 9 that is separated from the rest of the context by a wall. In contrast, the WGAN avoids placing poses far from the existing context (Figure 10C) without a physically based rule. This result further highlights the difficulty of handcrafting solutions to the pose generation problem, as these solutions need to effectively balance proxemics, spatial environmental constraints, and arbitrary conversational group sizes.

## 8 Discussion

### 8.1 Summary of Contributions

Our work introduced two approaches for generating poses for social robots in group conversations given spatial constraints and the pose of other group members. One approach formalizes key geometric properties of spatial behavior evident in conversational groups. In this Geometric approach, generating the location of a pose is formulated as an optimization problem, whose loss function penalizes divergence from the circular shape of the existing group formation, violations of personal space, and robot placement in nonfree environmental areas. The other, data-driven approach models expected spatial behavior with a WGAN. The inputs to the generator and discriminator networks are a map of the environment and a social interaction graph, where the graph nodes correspond to the pose features of existing interactants. Our novel architectures for the generator and discriminator rely on GNNs, which reason about spatial–orientational arrangements and proxemic relationships in a more implicit manner than our Geometric approach.

We evaluated our proposed methods on the Cocktail Party dataset with metrics based on desirable properties of conversational group formations. We chose for a baseline a pose generation method that does not consider environmental characteristics. Both of our approaches significantly outperformed the baseline method on metrics for maintaining the circular shape of the group and accounting for obstacles in the environment. This evaluation affirms the importance of considering environmental constraints in addition to interactants’ poses when generating spatial behavior for social agents.

The quantitative evaluation also informed model selection for a second evaluation, which compared our two pose generation approaches from a human perspective. Study participants assessed poses generated by our two proposed approaches in virtual scenes. With respect to an In Group measure, the Geometric approach generated superior poses for groups of three or four interactants, whereas the data-driven approach scored better for larger groups. The contrasting strengths of our two approaches further reinforce the complexity of pose generation in social applications: an optimal solution must respect spatial constraints from both the environment and other group members while also considering human expectations for behavior in a variety of scenarios.

In addition to the above contributions, this work explored using the proposed pose generation methods to simulate conversational groups of different sizes. We are excited about the potential of this application to enhance robotics simulations for HRI, like SEAN (Tsoi et al., 2020), as the proposed methods could be used as a practical mechanism to add human–robot social interactions to virtual environments. This could allow the community to further study social robot navigation (Mavrogiannis et al., 2021) or advance our understanding of proxemics and human perception of spatial patterns of behavior in HRI (Li et al., 2019; Connolly et al., 2021).

### 8.2 Limitations and Future Work

Our work is limited in several ways, which we consider avenues for future work. First, we did not find a clear winner between the proposed pose generation methods. The Geometric approach led to best quantitative metrics, but according to human ratings, it did not perform as well as the data-driven method with bigger groups. While we believe that in the long term the data-driven approach is more likely to succeed than the Geometric approach because it has more flexibility to reason about the intricacies of human spatial behavior, it is heavily dependent on the availability of significant amounts of realistic data. Thus, future work could explore creating better datasets for pose generation subject to environmental spatial constraints and reevaluate the WGAN on such datasets. One interesting idea in this respect is leveraging the Geometric approach to augment the training data used for the data-driven method.

Second, we focused on predicting a suitable pose for a robot given the location and orientation of interactants, but one could consider additional input features for the context in the future, such as motion data. Adding this information to the Geometric approach may require additional special considerations, but providing more input features to the data-driven method is easier. For example, we could adjust the architecture of the spatial–orientational GNN used in the generator and critic to take on more input features per interactant and thus allow the networks to reason about this additional information.

Third, our work is limited in that our evaluation of the proposed approaches considered simulated interactions only. We have not yet evaluated the methods on real-world human robot interactions. In the future, we would like to study the effectiveness of the proposed methods to enable robots to adapt their pose during situated group conversations, as interactants move or come and go. We would also like to explore using the proposed methods for enabling robots to join nearby group conversations subject to physical environmental constraints.

Fourth, we often assumed in this work that robots should behave in similar ways to humans. However, prior work suggests that robot embodiment may affect the way in which people interpret robot spatial behavior in HRI (Connolly et al., 2021). Thus, future work should investigate whether the proposed methods are suitable for different types of robots, especially those that are less anthropomorphic than Pepper.

## Data Availability

The datasets presented in this study can be found in online repositories. The names of the repository/repositories and accession number(s) can be found below: https://gitlab.com/interactive-machines/spatial_behavior/genff.

## Footnotes

1 Splitting the dataset in this manner minimized overlap between the training and testing data given the temporal correlation of the data.

2 The renderings were generated as in Connolly et al. (2021), using the Unity game engine (https://unity.com/), tools from the Social Environment for Autonomous Navigation (Tsoi et al., 2020), the Microsoft Rocketbox avatar library (Gonzalez-Franco et al., 2020), and an open-source version of Pepper’s Universal Robot Description File (http://wiki.ros.org/pepper_description).
