# Supplementary material for: Pose Generation for Social Robots in Conversational Group Formations
Source: Front Robot AI. 2022 Jan 17;8:703807. doi: 10.3389/frobt.2021.703807 (PMC8801779; doi:10.3389/frobt.2021.703807)
Supplement: Supplementary file 1 [file DataSheet1.pdf]

Supplementary Material

1 SIMULATING CONVERSATIONAL GROUP FORMATIONS

We created a large amount of training data for the WGAN using 3D environments from the iGibson simulator (Shen et al., 2020). More specifically, this data was created in 3 main steps. First, we automatically generated environment layouts with free and occupied space for 15 interactive environments from iGibson, as illustrated in Fig. S1a. The layouts were created by intersecting planes parallel to the ground with the 3D geometry of the environments. Second, we manually annotated the layouts to fill in occupied spaces and, using the layouts, created 15 maps for pose estimation that had labeled “free space”, occupied space by “short objects” and occupied space by “tall objects.” Third, we populated the maps with simulated groups as explained in the next Section.

1.1 A Rule-Based Approach to Create Circular Spatial Arrangements

We implemented a simple rule-based approach for creating circular formations typically observed during conversations. The algorithm took as input an environment map from iGibson. It output the poses for members of a simulated group in the map and a cropped section of the map around the group. The algorithm had six main steps:

1. Select a group size uniformly from the set $\{2, 3, 4, 5, 6\}$ – which we chose to mimic the group sizes observed in the Cocktail Party dataset (Zen et al., 2010).
2. Randomly chose a radius from 0.8-1.5 meters for the circular formation.
3. Choose a random unoccupied space in the environment as the center of the group’s circular formation.
4. Choose a random location for the group members along the circular formation such that interactants would not be too close to one another.
5. Decide if the group’s placement is valid by checking if a number of relevant locations for the group do not fall on occupied spaces of the map. The relevant locations included the midpoints between any combination of 2 group members (so that group members could potentially see each other), midpoints between any person and the center of their circular formation (so that all group members had access to the F-Formation o-space), and locations within a meter around any person in the group (to avoid placing interactants too close to objects).
6. If the group passed the above check, orient the members towards the center of their circular formation and output their poses along with the section of the environment map that surrounds them; otherwise, repeat the above steps until a successful group is created or a maximum number of attempts is reached.

Although the above approach could have been optimized in many ways, it was chosen for its simplicity given that simulated data only needed to be generated once. Example groups generated through this approach can be seen in Figure S1b (first column).

1.2 Simulated iGibson Dataset

We initially generated 34,405 simulated groups for training on the 15 iGibson environments. Group sizes were distributed as follows: 8445 groups were dyads, 7240 groups were triads, 6611 groups had 4 members, 6063 groups had 5 members, and 6046 groups had 6 members. Because these groups were perfect circular arrangments, we decided to slightly stretch them (horizontally or vertically) and rotate them (along with

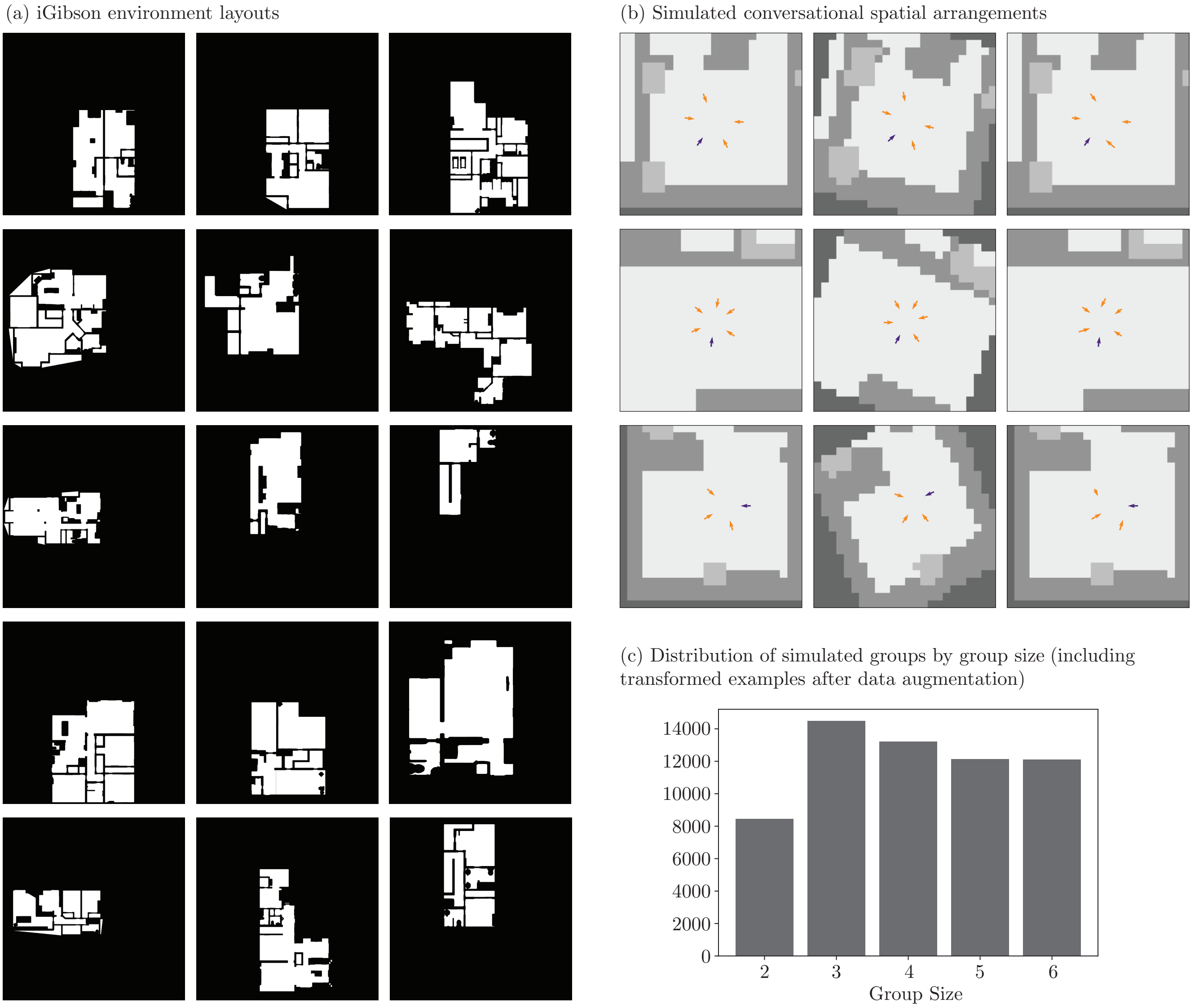

Figure S1. (a) The 15 iGibson environments from which we generated simulated groups. (b) Original simulated groups (first column), transformed group via stretching and rotation (second column), and the same original group after adding angular noise to the context (third column). (c) Final distribution of simulated groups by group size after data augmentation (stretching and rotations).

the environment) to add more variability to the simulated dataset. In particular, we transformed groups with 3 or more members, resulting in 25,960 additional training examples. Figure S1b (second column) shows example transformations applied to simulated groups. The final distribution of simulated groups (including those that were stretched and rotated) by group size is shown in Figure S1c.

As an additional type of data augmentation, we implemented a transformation for the iGibson data which added angular noise to the orientation of the context poses during training of the WGAN. The noise was sampled from a normal distribution with zero mean and a standard deviation corresponding to 20 degrees. Example results from this transformation can be seen in Figure S1b (third column). This transformation was not applied to Cocktail Party data during training because the latter data was already diverse in comparison to the perfect circular arrangements generated on the iGibson environments.

2 WGAN ARCHITECTURE

This section details the neural network architectures used for the generator G and discriminator (or critic) D of the proposed WGAN model. Both networks received as input the poses of the people in the context C and a cropped map of the environment around the context. The locations in the context poses were given relative to a coordinate frame whose origin was the average location of the context poses, corresponding to the center of the cropped map. This made the data translation invariant and facilitated training. Also, the generator received as input a latent variable $\mathbf{z}$, and the critic received an additional pose (from the true data distribution or from the generator). The location of the pose input to the critic was in the same coordinate frame as the context locations.

2.1 Generator Network

The generator network first processes its input graph with two GNNs, one in charge of reasoning about spatial-orientational information in the group's context and another one in charge of reasoning about proxemics information. The output of these two GNNs is then processed by a final multi-layered perceptron, as explained in Section 4.3 of the main paper. The sections below provide more implementation details for the generator network.

Spatial-Orientational GNN. The update function $\phi_1^v()$ described in the main paper is a multi-layer perceptron (MLP) and is implemented as outlined in Table S1 (left). The aggregate function $\rho_1^{v\rightarrow u}()$ is element-wise maximum.

Table S1. Architecture for the node update function of the Spatial-Orientational GNN. *Left:* Parameters for the generator. *Right:* Parameters for the critic. BN corresponds to batch norm.

Layer	Output dim.	Activation	BN
fc1	32	ReLU	Yes
fc2	64	ReLU	Yes
fc3	128	ReLU	Yes

Layer	Output dim	Activation	BN
fc1	32	ReLU	No
fc2	64	ReLU	No
fc3	128	ReLU	No

Proxemics GNN. This GNN first updates a node's features $\mathbf{v}_i = [x_i \ y_i \ \cos(\theta) \ \sin(\theta)]$ with the function $\mathbf{v}_i' = \phi_2^v(\mathbf{v}_i)$, which outputs a 2D tensor with a gaussian blob on the interactants location. The blob is generated using a normal distribution $\mathcal{N}(\cdot; \mu, I\sigma)$ with $\mu = [x_i \ y_i]^T$ and $\sigma = 0.21$ (as used for the personal space loss of the geometric approach). Then, the updated node features are aggregated into a feature $\bar{\mathbf{v}}'$ using element-wise summation. Finally, the global attribute of the input graph is updated using $\mathbf{u}' = \phi_2^u(\bar{\mathbf{v}}', \mathbf{u})$. The function $\phi_2^u()$ is implemented as a convolutional neural network (CNN) with zero padding, as detailed in Table S2, and with a final flatten layer.

Table S2. Architecture for the global feature update function of the Proxemics GNN used in the generator. BN corresponds to batch norm.

Layer	Channels Out	Kernel	Stride	Padding	Activation	BN
conv1	8	3×3	1	1	ReLU	True
maxpool1	8	2×2	2	0	–	–
conv2	32	3×3	1	1	ReLU	True
maxpool2	32	2×2	2	0	–	–
conv3	64	3×3	1	1	ReLU	True

Final Multi-Layer Perceptron. The final multi-layer perceptron of the generator is composed of three fully connected layers, as detailed in Table S3 (left). The last two elements of the 4D output of the MLP are finally applied a hyperbolic tangent transformation to constraint them to $(-1, 1)$ because they represent the $\cos(\theta)$ and $\sin(\theta)$ of the output pose.

Table S3. Architecture for the final MLP of the WGAN networks. *Left:* Parameters for the generator. *Right:* Parameters for the critic. BN is batch norm.

Layer	Output dim.	Activation	BN
fc1	1024	ReLU	No
fc2	512	ReLU	No
fc3	4	–	No

Layer	Output dim	Activation	BN
fc1	1024	ReLU	No
fc2	512	ReLU	No
fc3	1	–	No

2.2 Critic Network

The critic network is similar to the generator network described previously, except that its input graph has as global attribute the environment map only (without information about a latent variable $\mathbf{z}$) and the critic receives an additional input: a pose from the true data distribution or output by the generator, which is processed in a third parallel stream to the GNNs. The sections below provide more implementation details for each component of the critic network.

Spatial-Orientational GNN. The critic's Spatial-Orientational GNN is the same as for the generator, except that its node update function does not use batch normalization (BN) because BN can make it harder for the critic to converge, as discussed in (Gulrajani et al., 2017). Table S1 (right) details the parameters of the critic's node update function.

Proxemics GNN. The critic's Proxemics GNN is also the same as for the generator, except that the global attribute update function, which is implemented as a CNN, does not use batch norm.

Pose Multi-Layer Perceptron. The pose input to the critic is transformed with a series of fully collected layers, as detailed in Table S4.

Table S4. Architecture of the multi-layer perceptron that transforms poses input to the critic network. BN corresponds to batch norm.

Layer	Output dim.	Activation	BN
fc1	32	ReLU	No
fc2	64	ReLU	No

Final Multi-Layer Perceptron. The critic concatenates the outputs of its GNNs and the pose MLP and then transforms the resulting feature vector through another MLP, outlined in Table S3 (right).

3 ADDITIONAL QUANTITATIVE RESULTS FOR THE DATA-DRIVEN METHOD

In addition to the results presented in the main paper for the WGAN (Section 5), we also studied the performance of other variations for the data-driven model using the proposed quantitative metrics. These variations are described below:

WGAN with increased distribution size. We chose 36 samples for the models that computed distributions in the main paper because this number of samples reasonably covered the area around the context for the geometric approach. Howevever, we were curious about whether more samples could benefit the WGAN and, thus, we evaluated it when running the generator 576 times. The results are presented in Table S5. In comparison to Table 1 in the main paper, the increased distribution size had minimal effect on performance.

Table S5. Results on the Cocktail Party test set with a distribution of 576 samples from which we chose the biggest mode as final output. Each row shows $\mu \pm \sigma$ for the metrics described in the main paper (lower is better). "(iG)" models were trained on simulated data using iGibson environment maps, "(CP)" indicates training with Cocktail Party train data, and "(iG,CP)" corresponds to pretraining with simulated data and then finetuning on Cocktail Party train data.

	Method	Circ. Fit	Not Free	Per. Space	Int. Space	Center Dist.	Occ. Other	Is Occ.
1	WGAN (iG)	0.34 ± 0.28	0.06 ± 0.22	0.37 ± 0.64	0.10 ± 0.31	0.45 ± 0.14	0.05 ± 0.28	0.00 ± 0.05
2	WGAN (CP)	0.29 ± 0.23	0.02 ± 0.13	0.71 ± 0.71	0.30 ± 0.48	0.46 ± 0.12	0.11 ± 0.39	0.05 ± 0.21
3	WGAN (iG, CP)	0.31 ± 0.23	0.03 ± 0.14	0.66 ± 0.63	0.22 ± 0.41	0.45 ± 0.11	0.11 ± 0.31	0.02 ± 0.13

WGAN with combined map for tall and short obstacles. Because the geometric approach only has information about free and occupied space, we tested training the WGAN with a similar configuration. That is, we merged the two channels of the map input to the WGAN, which represented occupancy by tall and short objects, into a single map with occupied and free space information. The results for this test are presented in Table S6. In general, the performance was similar to the WGAN that used a two-channel map, as described in the main paper. Thus, we primarily evaluated the WGAN with two-channel maps in this work, which more explicitly described obstacles in the environment. Worth noting, though, in some cases the model trained with combined maps and only on simulated groups generated poses outside the input map, resulting in a higher Circ. Fit metric than the results in Table 1.

Table S6. Results on the Cocktail Party test set with combined environment channels. Each row shows $\mu \pm \sigma$ for the metrics described in the main paper (lower is better). Models without * output a single pose, whereas those with * output a distribution of 36 poses from which we chose the biggest mode as final output. "(iG‡)" models were trained on simulated data using iGibson environment maps (without data augmentation), "(CP)" indicates training with Cocktail Party train data, and "(iG‡,CP)" corresponds to pretraining with simulated data and then finetuning on Cocktail Party train data.

	Method	Circ. Fit	Not Free	Per. Space	Int. Space	Center Dist.	Occ. Other	Is Occ.
1	WGAN (iG‡)	0.52 ± 1.24	0.05 ± 0.21	0.47 ± 0.60	0.14 ± 0.35	0.47 ± 0.45	0.09 ± 0.37	0.03 ± 0.16
2	WGAN (CP)	0.30 ± 0.25	0.01 ± 0.10	0.67 ± 0.70	0.27 ± 0.48	0.45 ± 0.12	0.10 ± 0.35	0.04 ± 0.20
3	WGAN (iG‡,CP)	0.29 ± 0.23	0.01 ± 0.09	0.72 ± 0.68	0.29 ± 0.48	0.40 ± 0.13	0.09 ± 0.39	0.06 ± 0.23
4	WGAN* (iG‡)	0.51 ± 1.24	0.06 ± 0.22	0.49 ± 0.60	0.14 ± 0.36	0.47 ± 0.45	0.07 ± 0.31	0.02 ± 0.15
5	WGAN* (CP)	0.31 ± 0.25	0.02 ± 0.13	0.68 ± 0.70	0.28 ± 0.50	0.45 ± 0.12	0.09 ± 0.28	0.03 ± 0.16
6	WGAN* (iG‡,CP)	0.30 ± 0.23	0.01 ± 0.09	0.71 ± 0.67	0.29 ± 0.46	0.40 ± 0.12	0.11 ± 0.42	0.05 ± 0.22

WGAN with personal space loss. In initial experiments, we also considered a modified version of the WGAN in which the generator was trained with an additional component for its loss which penalized for output poses that violated personal space. This component was implemented in the same manner as ℓ_p in eq. (4) in the main paper. This means that the loss for the WGAN was:

$$\min_G \max_D \mathbb{E}_{\mathbf{p}\sim\mathbb{P}_r}[D(\mathbf{p}|C,M)] - \mathbb{E}_{\bar{\mathbf{p}}\sim\mathbb{P}_g}[D(\bar{\mathbf{p}}|C,M)] + \lambda\mathbb{E}_{\bar{\mathbf{p}}\sim\mathbb{P}_g}\ell_p(\bar{\mathbf{p}}) \quad \text{(S1)}$$

We set $\lambda = 0.1$ based on validation performance, and obtained the results shown in Table S7 using the original iGibson simulated groups (without data augmentation in the form of stretching, rotations, nor angle noise). We found that the addition of the personal loss to the generator reduced in some cases violations to intimate spaces in comparison to not adding the loss and training the model on the iGibson data without

Table S7. Results on the Cocktail Party test set. Each row shows $\mu \pm \sigma$ for the metrics described in the main paper (lower is better). Models without $*$ output a single pose, whereas those with $*$ output a distribution of 36 poses. The $+\ell_p$ marker indicates that the WGAN generator was trained with a penalty for violating personal space (i.e., with personal loss). "(iG‡)" models were trained on simulated data using iGibson environment maps (without data augmentation), "(CP)" indicates training with Cocktail Party train data, and "(iG‡,CP)" corresponds to pretraining with simulated data and then finetuning on Cocktail Party train data.

	Method	Circ. Fit	Not Free	Per. Space	Int. Space	Center Dist.	Occ. Other	Is Occ.
1	WGAN+ℓ_p (iG‡)	0.34 ± 0.28	0.03 ± 0.16	0.41 ± 0.63	0.12 ± 0.34	0.42 ± 0.13	0.05 ± 0.41	0.01 ± 0.11
2	WGAN+ℓ_p (CP)	0.32 ± 0.23	0.02 ± 0.13	0.70 ± 0.68	0.29 ± 0.48	0.44 ± 0.12	0.11 ± 0.31	0.05 ± 0.21
3	WGAN+ℓ_p (iG‡,CP)	0.31 ± 0.23	0.04 ± 0.17	0.66 ± 0.64	0.24 ± 0.45	0.44 ± 0.12	0.08 ± 0.27	0.03 ± 0.16
4	WGAN*+ℓ_p (iG‡)	0.35 ± 0.29	0.03 ± 0.16	0.42 ± 0.62	0.13 ± 0.34	0.42 ± 0.13	0.05 ± 0.33	0.02 ± 0.13
5	WGAN*+ℓ_p (CP)	0.31 ± 0.23	0.02 ± 0.13	0.71 ± 0.66	0.31 ± 0.49	0.44 ± 0.12	0.14 ± 0.43	0.03 ± 0.18
6	WGAN*+ℓ_p (iG‡,CP)	0.31 ± 0.23	0.04 ± 0.16	0.65 ± 0.62	0.22 ± 0.43	0.43 ± 0.12	0.05 ± 0.22	0.03 ± 0.18

data augmentation. However, the data augmentation allowed us to obtain similar or better performance without the personal space loss, as can be seen by comparing the results in Table S7 with those in the main paper. We are excited about this result because the WGAN encoded important properties of F-Formations without a hand-crafted loss specifically designed for our problem domain. This flexibility means that the WGAN could be applied to other related problems in the future without major modifications, e.g., predicting poses for robots in other interactions like queues or side-by-side walking.

4 SURVEY USED FOR THE HUMAN EVALUATION

The human evaluation was carried out using Qualtrics online survey software. We organized the survey into 4 main sections:

1. Demographics section, e.g., with questions about age, gender, "how often do you play video games?", and "how often do you interact or work with a robot".

2. Practice section, which showed a robot in two scenes to familiarize them with the task of providing In Group ratings. First, the robot was shown using a ground truth pose from the Cocktail Party dataset. Second, it was shown having a bad orientation, as described in the main paper. Figure S2 shows the top-down renderings used for this section of the study. The presentation of the practice scenes within the survey was the same as for the evaluation scenes that followed.

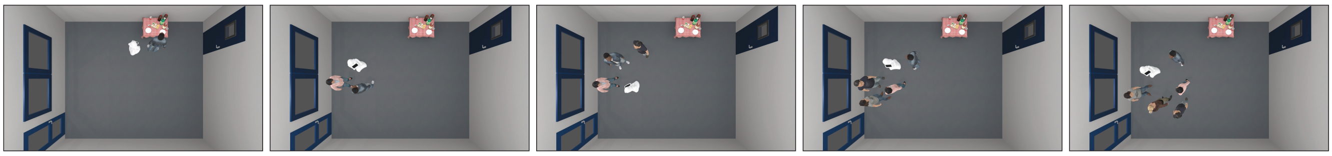

(a) Ground truth poses from the Cocktail Party data

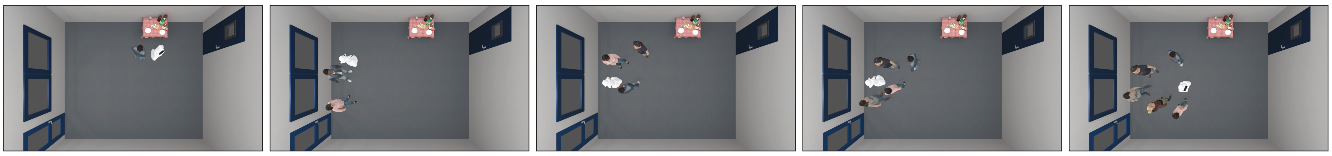

(b) Bad poses (the robot was oriented away from the group)

Figure S2. Practice top-down renderings used as practice in the survey. From left to right, the images show Group Sizes of 2, 3, 4, 5, and 6 interactants (including the robot).

3. Evaluation section, where the participants were asked to rate the pose of the robot in twenty scenes. Half of the scenes had the robot positioned as directed by the model-based approach; the other half used poses output by the data-driven method. The participants did not know which method was used in each rendering. Also, the order of the 20 scenes was randomized per participant to avoid potential ordering effects. An example page of this section of the survey is shown in Figure S3. All the top-down view renderings used in the evaluation are shown in Figures S4, S5, S6, S7 and S8.

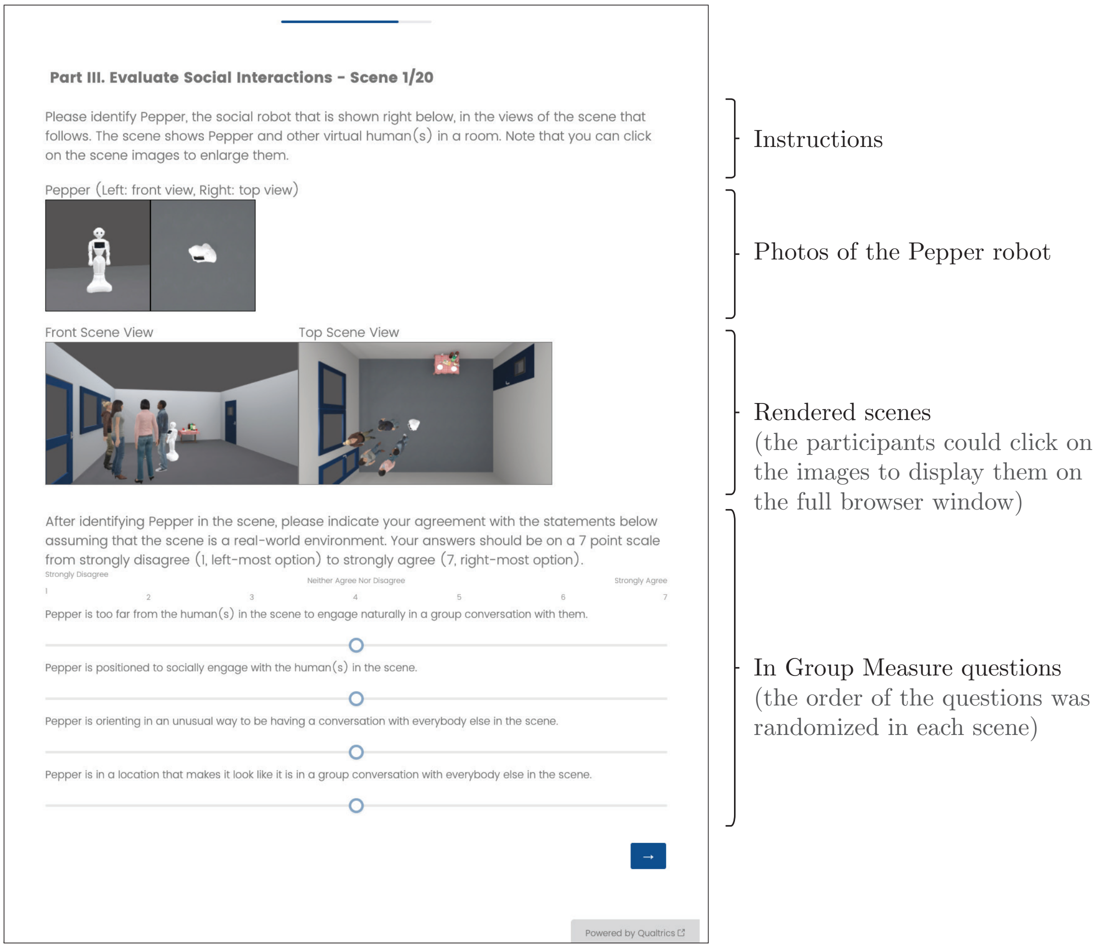

Figure S3. Example evaluation page from the survey.

4. Final feedback section, which asked the participants to answer the question: "If you thought that the survey was difficult to complete for any particular reason, please explain below in detail what kind of difficulties you encountered with the survey." This question helped clarify the presentation of the instructions in pilots.

5 DETAILED STATISTICS FOR IN GROUP RATINGS

For every scene in the survey used for the human evaluation, the participants provided their agreement with the four statements shown in Figure S3. The statements were: (1) Pepper is too far from the human(s) in the scene to engage naturally in a group conversation with them; (2) Pepper is in a location that makes it look like it is in a group conversation with everybody else in the scene; (3) Pepper is positioned to socially engage with the human(s) in the scene; and (4) Pepper is orienting in an unusual way to be having a conversation with everybody else in the scene. These statements composed the In Group measure described in the main paper. Their means, standard deviations, and correlations are shown in Table S8.

Table S8. Descriptive statistics and correlations for the In Group statements. Ratings for each statement were obtained using a 7-point responding format from "strongly disagree" (1) to "strongly agree" (7). *(R)* indicates that the ratings were reversed before computing the descriptive statistics and correlations. Also, *** indicates that the pair-wise correlation was statistically significant with $p < 0.001$.

Statement	N	M	STD	1	2	3	4
1. Pepper is too far from the human(s) in the scene to engage naturally in a group conversation with them *(R)*	1,188	5.37	1.86	–			
2. Pepper is in a location that makes it look like it is in a group conversation with everybody else in the scene	1,188	4.75	1.95	0.48***	–		
3. Pepper is positioned to socially engage with the human(s) in the scene	1,188	4.88	1.93	0.48***	0.84***	–	
4. Pepper is orienting in an unusual way to be having a conversation with everybody else in the scene *(R)*	1,188	4.64	2.07	0.39***	0.55***	0.56***	–

REFERENCES

Gulrajani, I., Ahmed, F., Arjovsky, M., Dumoulin, V., and Courville, A. C. (2017). Improved training of wasserstein gans. In *Advances in neural information processing systems*. 5767–5777

Shen, B., Xia, F., Li, C., Martín-Martín, R., Fan, L., Wang, G., et al. (2020). iGibson, a Simulation Environment for Interactive Tasks in Large Realistic Scenes. *arXiv preprint arXiv:2012.02924*

Zen, G., Lepri, B., Ricci, E., and Lanz, O. (2010). Space speaks: towards socially and personality aware visual surveillance. In *Proceedings of the 1st ACM international workshop on Multimodal pervasive video analysis*. 37–42

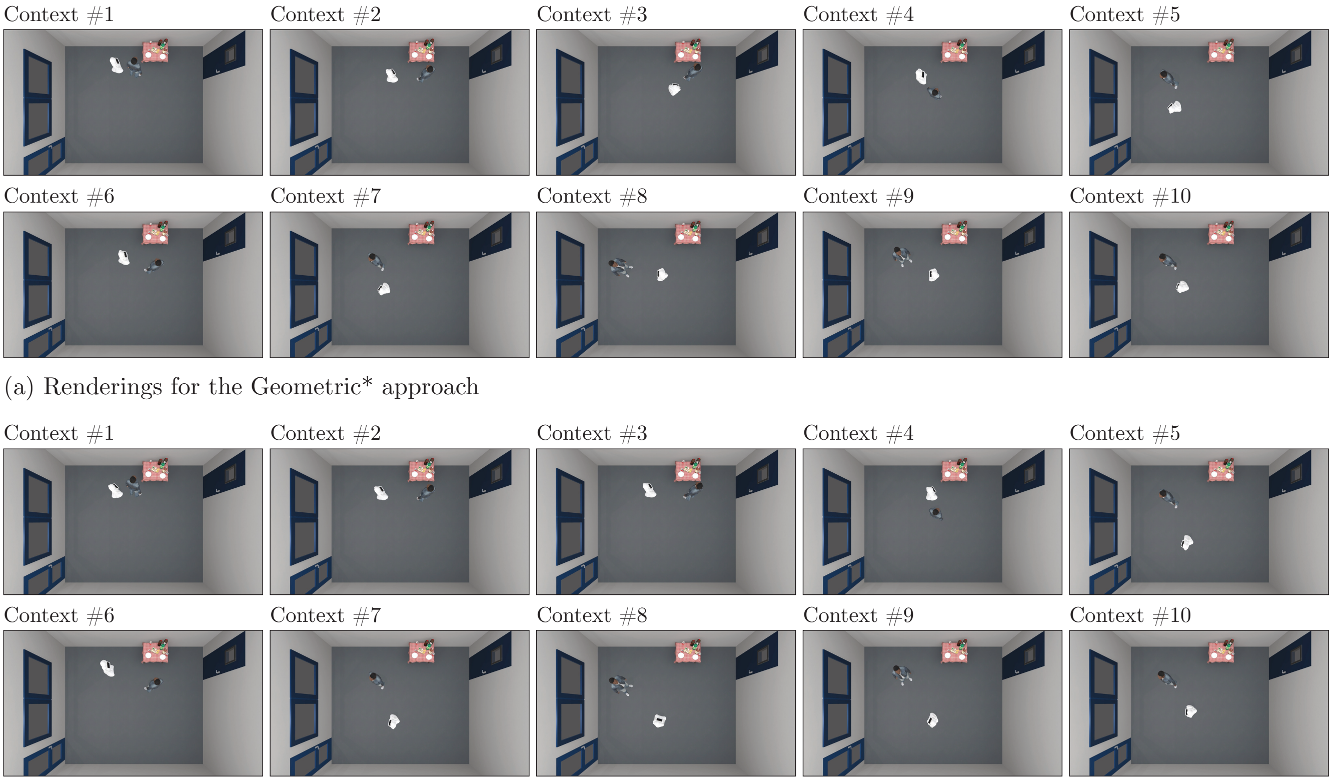

(a) Renderings for the Geometric* approach

(b) Renderings for the WGAN* approach

Figure S4. Top-down renderings for a Group Size of 2. The renderings were used in our human evaluation.

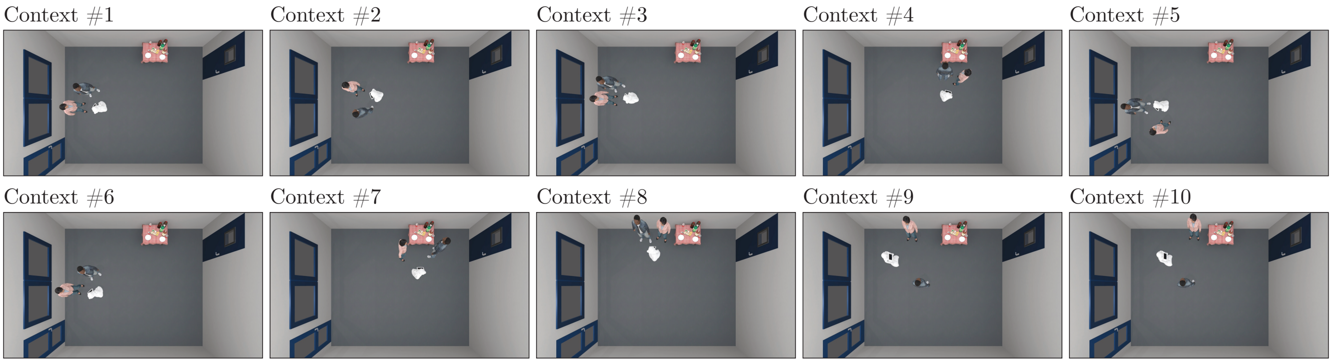

(a) Renderings for the Geometric* approach

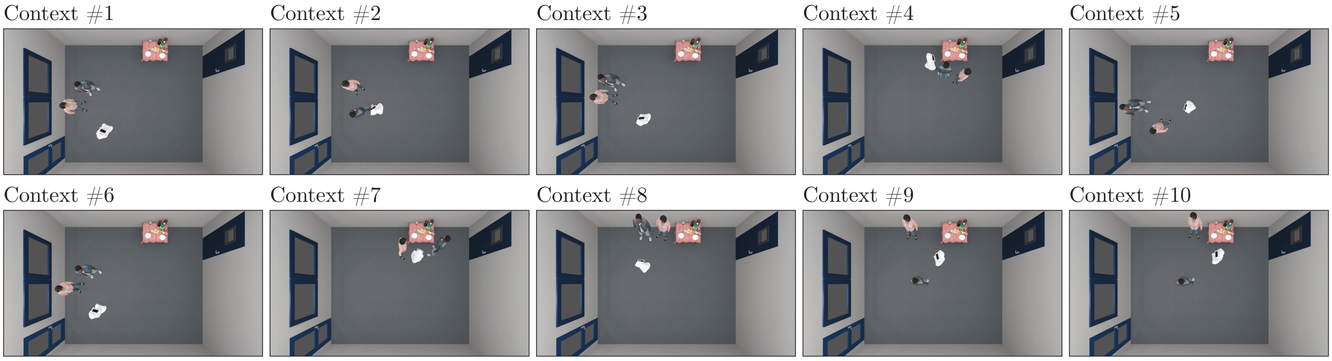

(b) Renderings for the WGAN* approach

Figure S5. Top-down renderings for a Group Size of 3. The renderings were used in our human evaluation.

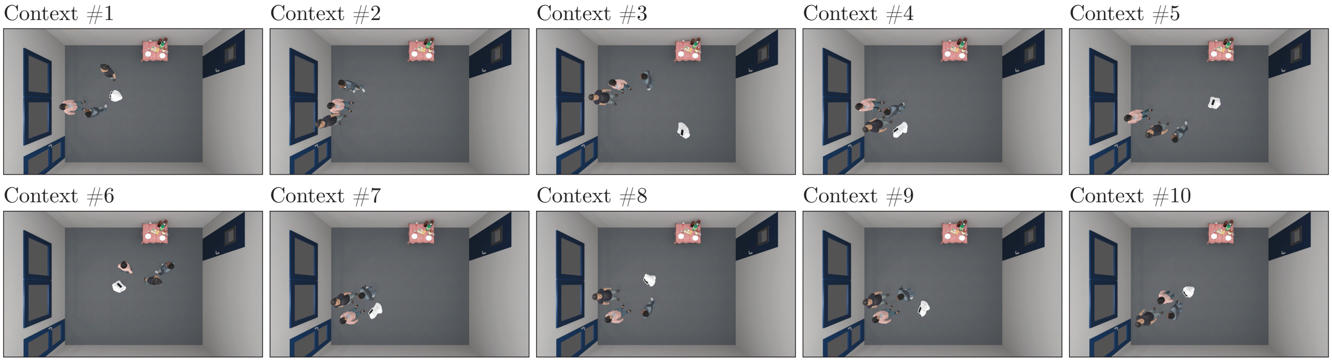

(a) Renderings for the Geometric* approach

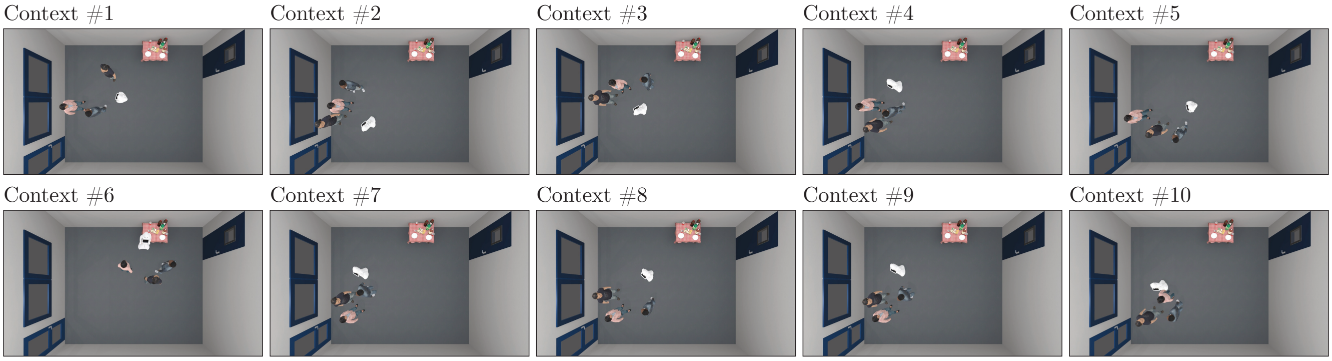

(b) Renderings for the WGAN* approach

Figure S6. Top-down renderings for a Group Size of 4. They were used in our human evaluation, except for the Context #2 prediction by the Geometric* approach (which placed the robot outside of the room).

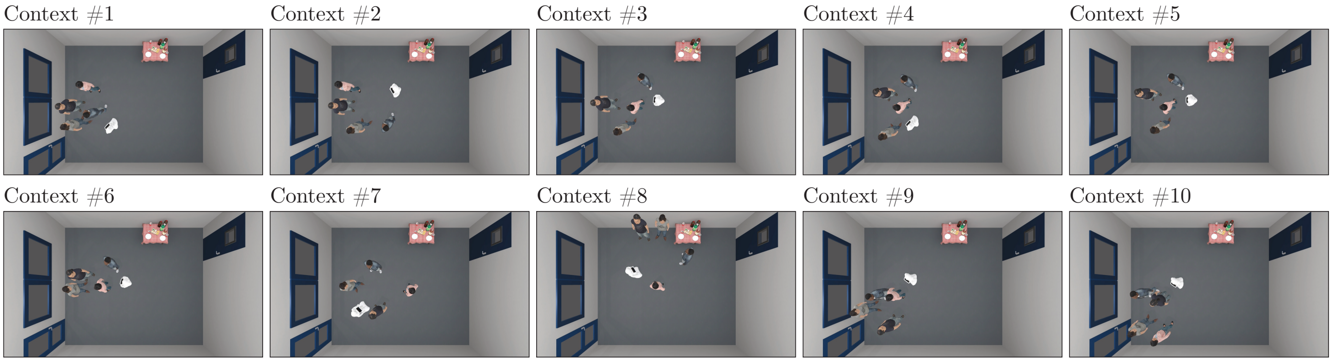

(a) Renderings for the Geometric* approach

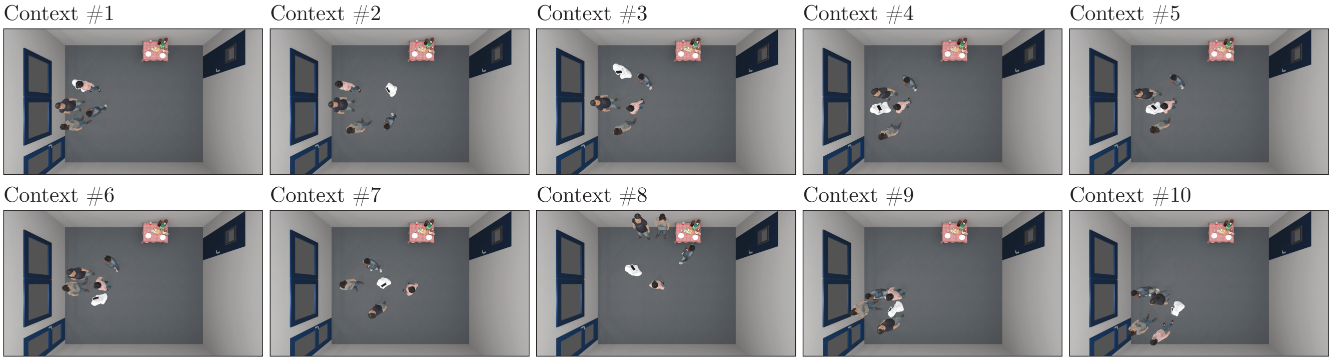

(b) Renderings for the WGAN* approach

Figure S7. Top-down renderings for a Group Size of 5. The renderings were used in our human evaluation.

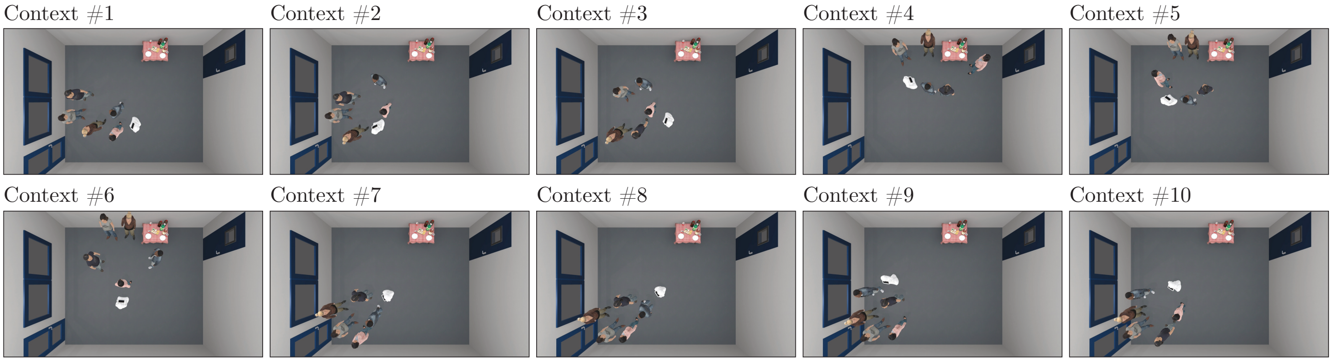

(a) Renderings for the Geometric* approach

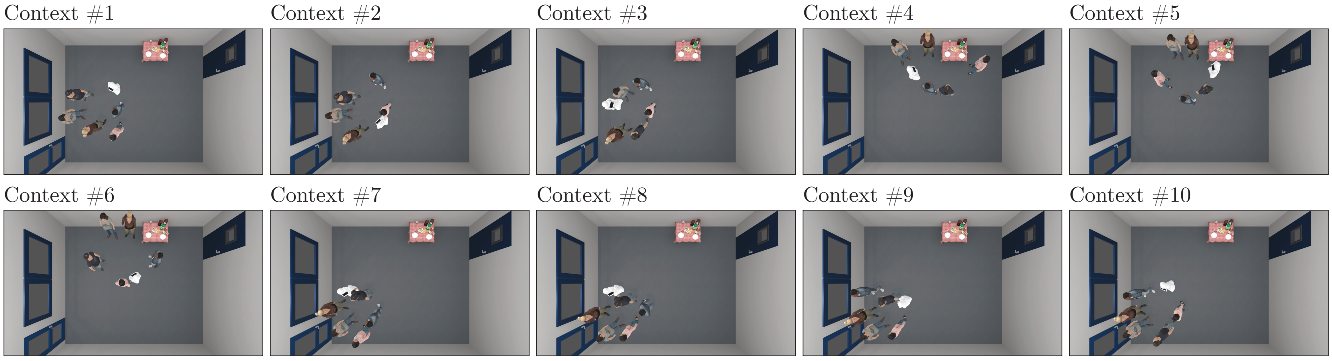

(b) Renderings for the WGAN* approach

Figure S8. Top-down renderings for a Group Size of 6. The renderings were used in our human evaluation.
